# Evaluating the potential implications of canadian front-of-pack labelling regulations in generic and branded food composition databases

**DOI:** 10.1186/s12889-022-14269-4

**Published:** 2022-10-06

**Authors:** Christine Mulligan, Jennifer J. Lee, Laura Vergeer, Mavra Ahmed, Mary R. L’Abbé

**Affiliations:** 1 Department of Nutritional Sciences, Temerty Faculty of Medicine, University of Toronto, Medical Sciences Building, Room 5368, 1 King’s College Circle, M5S 1A8 Toronto, ON Canada; 2 Joannah & Brian Lawson Centre for Child Nutrition, University of Toronto, University of Toronto, 1 King’s College Circle, M5S 1A8 Toronto, ON Canada

**Keywords:** Front-of-pack labelling, Nutrition symbols, Labelling regulations, Food composition, Pre-packaged foods, Food supply system, Nutrients-of-concern

## Abstract

**Background:**

Canada proposed the implementation of mandatory front-of-pack (FOP) labelling regulations, whereby foods meeting or exceeding thresholds for nutrients-of-concern (i.e., total sugars, saturated fat, sodium) must display a ‘high-in’ FOP symbol (FOP). The objective of the study was to evaluate the potential implications of the proposed regulations using Canadian generic and branded food composition databases.

**Methods:**

A generic food composition database of products consumed by Canadians, Canadian Nutrient File (CNF) 2015 (n = 3,677), and a branded food composition database of packaged foods and beverages, Food Label Information Program (FLIP) 2017 (n = 17,521), were used to evaluate the number and proportion of foods that would display a FOP symbol based on the details of the proposed FOP labelling regulations published in 2018.

**Results:**

Overall, 35.5% (n = 1,306) of products in CNF 2015 and 63.9% (n = 11,193) of products in FLIP 2017 would display a FOP symbol for at least one nutrient-of-concern exceeding proposed thresholds. Soups, Combination Dishes, and Desserts categories in CNF 2015 and Combination dishes, Soups, and Meats categories in FLIP 2017 would have the highest proportion of products that would display a FOP symbol. Although displaying a FOP symbol for one nutrient was most common in both CNF 2015 (n = 992; 27.0%) and FLIP 2017 (n = 7,296, 41.6%), the number (i.e., 0–3) and type (i.e., saturated fat, sodium, total sugar) of nutrients displayed varied by food category.

**Conclusion:**

While the generic database, containing both packaged and unpackaged foods, revealed a low prevalence of foods that would display a FOP symbol, the branded database showed that the proposed FOP labelling regulations would identify over 60% of packaged foods with excess contents of nutrients-of-concern. Considering the high prevalence of packaged foods in Canada that would meet or exceed the thresholds of nutrients-of-concern, the proposed FOP labelling regulations should be implemented in a timely manner to help consumers easily identify foods high in nutrients-of-concern and encourage manufacturer-driven product reformulations.

**Supplementary Information:**

The online version contains supplementary material available at 10.1186/s12889-022-14269-4.

## Introduction

Diet-related non-communicable diseases, including diabetes and cardiovascular diseases, are one of the major causes of disability and pre-mature mortality globally [1] and in Canada [2]. In the last few decades, front-of-pack (FOP) labelling has been used as a public health strategy to improve dietary patterns on a population level. FOP labelling refers to a simple, interpretative text and/or symbol-based label to communicate nutritional information about a product, which can help consumers easily and correctly identify the healthfulness of food products. FOP labeling has been shown to increase consumers’ awareness of the nutritional value of foods and nudge consumers towards healthier decisions [3, 4]. FOP labelling can also improve the nutritional quality of the food supply through manufacturer-driven product reformulation or through the introduction of new products with lower levels of nutrients-of-concern [5–7].

In 2018, Health Canada proposed FOP labelling regulations in *Canada Gazette I* to facilitate healthier and informed food choices by consumers and to improve the overall healthfulness of Canada’s food environment [8]. As of November 2021, the regulations are awaiting potential amendments and publication in *Canada Gazette II* before they can be implemented. The currently proposed FOP labelling regulations would mandate that foods meeting or exceeding recommended thresholds for nutrients-of-concern (i.e., total sugars, saturated fat, sodium) be required to display a ‘high-in’ FOP symbol [9]. Studies on Canadian consumers’ responses to FOP labelling in the form of a ‘high-in’ FOP symbol have shown the potential decreases in preference for and purchasing intentions of foods and beverages with a FOP symbol [10–15]. Although branded food composition databases in Canada have been used to evaluate the levels of specific nutrients-of-concern [16–20], and for testing the potential impact of the proposed FOP labelling regulations on a subsample of products from specific food companies [18], an examination of the FOP labelling regulations using on a broader sample of food and beverage products available to Canadian consumers is warranted. Given that Canada’s national dietary intake survey (i.e., Canadian Community Health Survey) is linked to a generic food composition database, the appropriateness of the database for assessing policy implications and subsequent outcomes needs to be examined. Therefore, the objective of the study was to evaluate the proportion of food products that would be required to display a FOP symbol according to the regulations proposed in *Canada Gazette I* in 2018 [9] using both a generic and a branded Canadian food composition database.

## Materials and methods

### Study Design

A cross-sectional analysis of foods commonly consumed by Canadians and the packaged retail food supply was conducted using the Canadian Nutrient File (CNF) 2015 database and the University of Toronto’s Food Label Information Program (FLIP) 2017 database, respectively. Foods were evaluated using the proposed FOP labelling regulations published in *Canada Gazette I* by Health Canada in 2018 [9].

### Canadian nutrient file (CNF) 2015

CNF is a database of foods commonly consumed by Canadians, including fresh and packaged foods available on the Canadian market, as well as home made meal products, with data available for up to 152 nutrients [21]. The nutrient information is derived from the United States Department of Agriculture National Nutrient Database for Standard Reference with modifications for Canadian levels of fortification and regulatory standards, as necessary; Canadian-specific foods; and other Canadian commodity data from some brand name foods. The nutrient composition in CNF foods reflects the average nutrient composition of foods derived from brands of similar products or varieties of foods from various producers [21]. The CNF 2015 database was downloaded for analysis via from Health Canada’s publicly available website. All products were categorized into Health Canada’s Table of Reference Amounts for Food (TRA) categories, representing the amount of food typically consumed in one sitting, which serves as the basis for determining serving sizes in the Nutrition Facts Table [22]. Health Canada’s TRA categories consist of 23 major and 171 minor categories.

A total of 6,904 products were available in the CNF database. Meal products created using common preparation methods reported by Canadians (e.g., homemade lasagna; n = 3,169) and products not regulated under the Food and Drugs Regulations (e.g., alcoholic beverages, nutritional supplements; n = 58) were excluded from the analysis. The final analytic sample from CNF included was 3,677 products.

### Food label information program (FLIP) 2017

The Food Label Information Program (FLIP) is a branded food database developed and maintained by the University of Toronto, details for which have been published elsewhere [18, 23–28]. Briefly, FLIP 2017 data was collected between May and September 2017 and contains nutritional information for 17,671 unique packaged food and beverage products from top Canadian food retailers (Loblaws, Sobeys and Metro), representing approximately 64% of the retail market share at the time of collection. Information contained in FLIP includes product name, company/brand, Nutrition Facts table information, ingredients list, universal product code, and photos of all sides of the package. However, given the nature of the FLIP database, fresh or unpackaged foods (e.g., fresh fruits and vegetables, and raw single ingredient meats) are not included. All products were categorized into Health Canada’s TRA categories for analysis.

A total of 17,521 packaged foods and beverages from FLIP 2017 were analyzed, following the exclusion of 150 products (i.e., foods for special dietary use (e.g., meal replacement bars), non-caloric sweetening agents (e.g., stevia), and products with missing nutritional information).

### Canadian front-of-Pack (FOP) labelling regulations

The proposed Canadian FOP labelling regulations released in *Canada Gazette I* [9] by Health Canada, the department responsible for federal food and nutrition regulations, were used to identify food/beverage products in the CNF 2015 and FLIP 2017 databases that would be mandated to display a FOP symbol. The proposed regulations would mandate that all food and beverage products display FOP labelling in the form of a ‘high in’ FOP symbol, if a product meets or exceeds thresholds for three nutrients-of-concern: total sugars; saturated fat; and sodium. Products are assessed under each nutrient threshold individually, so a product can display a FOP symbol for as little as 1 nutrient to as many as 3 nutrients, depending on its levels of nutrients-of-concern.

The threshold levels would be set based on the percent Daily Value (%DV) per serving size or reference amounts, as per TRA, for each nutrient and the product type (15% DV for foods/beverages and 30% DV for meals). The %DV would be set for two different age groups (i.e., children over 4 years of age and adults, and 1–4 year-old children) based on the Recommended Daily Intakes and the reference standards for nutrients [29]. For food and beverage products with serving size or reference amounts less than 50 g or mL, a standard 50 g would be used as a reference amount to assess the levels of nutrients-of-concern. **Supplementary Table 1** shows the summary of the thresholds for each nutrient-of-concern by product type and age group as outline in *Canada Gazette I* [9].

The proposed FOP labelling regulations included three categories of food/beverage products that would be exempted from displaying a FOP, regardless of the product’s nutrient content. First, products generally exempted from displaying a Nutrition Facts table would be exempted (e.g., fresh fruits and vegetables, raw single-ingredient meats) as the lack of Nutrition Facts table prevents consumers from seeking additional nutrition information at the point of purchase. Second, products that have been shown to have health benefits (e.g., fruits and vegetables, nuts, and unflavoured milk) would be exempted as a FOP symbol may provide conflicting health information. Third, products that are well-known sources of the target nutrients would be exempted as a FOP symbol would provide redundant information; these foods include table sugar, honey, maple syrup, salt and flavoured salts [9].

Each food product was manually evaluated against the exemption criteria by CM and JJL and verified by MA. Any disagreement was discussed until group consensus was achieved. FOP labelling thresholds for each nutrient-of-concern by product type and age group was applied using an algorithm to determine whether a product would meet or exceed the thresholds for each examined nutrient (i.e., display a FOP symbol).

### Statistical analysis

The number and proportion of products in CNF 2015 and FLIP 2017 that would be required to display a FOP symbol were calculated overall and by TRA major and minor categories. The number (i.e., 0–3) and type (i.e., total sugars, saturated fat, sodium) of ‘high in’ nutrients that products would be required to display using a FOP symbol were analyzed overall and by TRA major and minor categories.

## Results

### CNF 2015

**Fig. 1:**
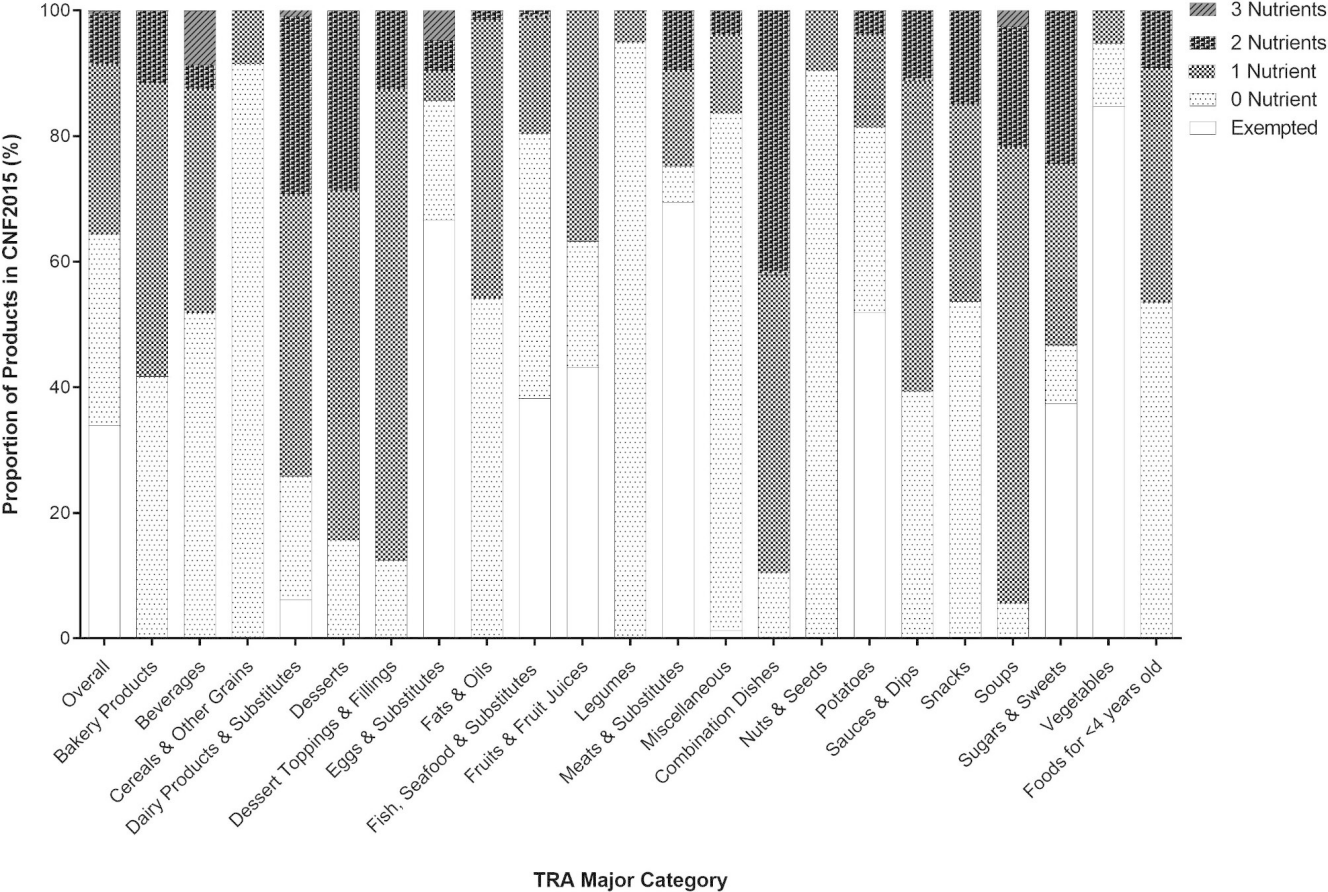
Proportion of food & beverage products in the Canadian Nutrient File (CNF) 2015 that would be affected by the proposed Front-of-Pack (FOP) Labelling Regulations in Canada by Table of Reference Amounts (TRA) major food category. n = 3,677. A total of 354 (9.6%), 62 (1.7%), and 20 (0.5%) products were missing values for total sugars, saturated fats, and sodium, respectively, and were removed from analyses. Food and beverage products were divided into 5 categories: Exempted (i.e., exempted from FOP labelling regulations), 0 Nutrient (i.e., would not display a FOP symbol for having nutrient levels below the threshold levels), and 1–3 Nutrients (i.e., would display a FOP symbol for meeting or exceeding threshold levels for 1–3 nutrient(s) of concern)

Using the threshold levels in the proposed Canadian FOP labelling regulations, 64.5% of products (n = 2,371) would not display a FOP symbol, while 35.5% of products (n = 1,306) would display a FOP symbol. Among products that would not display a FOP symbol, 52.6% of products (n = 1,247/2,371) would be exempted from the FOP labelling regulations (i.e., falls under an exemption category) and 47.4% of products (n = 1,124/2,371) would not exceed the nutrient thresholds for total sugars, saturated fat, or sodium. Among products that would display a FOP symbol, 76.0% of products (n = 992/1,306) would indicate one ‘high in’ nutrient, 22.1% of products (n = 289/1,306) would indicate two ‘high in’ nutrients, and 1.1% of products (n = 15/1,306) would indicate ‘high in’ all three nutrients. Figure 1 and **Supplementary Tables 2–3** show the proportion of products in CNF 2015 categorized by the exemption criteria and the number of nutrients that products would display a FOP symbol based on the proposed Canadian FOP labelling regulations, presented by overall and by TRA major and minor food categories.

The top 3 TRA categories with products that would be exempted from displaying a FOP symbol were Vegetables (84.7%; n = 326), Meats & Substitutes (69.5%; n = 600), and Eggs & Substitutes (66.7%, n = 14). The top 3 TRA categories with products that would not display a FOP symbol for being below the threshold levels for nutrients-of-concern were Cereals & Other Grain Products (91.5%; n = 172), Legumes (95.0%; n = 113), and Nuts & Seeds (90.5%; n = 86). The top 3 TRA categories with products that would display a FOP symbol for one ‘high-in’ nutrient were Soups (72.7%; n = 141), Dessert Toppings & Fillings (75.0%; n = 6), and Desserts (55.7%; n = 39). The top 3 TRA categories with products that would display a FOP symbol for two ‘high-in’ nutrients were Combination Dishes (42.1%; n = 8), Dairy Products & Substitutes (28.2%; n = 59), and Desserts (28.6%; n = 20). The top 3 TRA categories with products that would display a FOP symbol for three ‘high-in’ nutrients were Beverages (8.9%; n = 7), Eggs & Substitutes (4.8%, n = 1), and Soups (2.6%, n = 5).

**Supplementary Table 4** shows the proportion of products in CNF 2015 categorized by the nutrient type on a FOP symbol that products would display based on the proposed Canadian FOP labelling regulations by TRA major and minor categories. Among products that would display a FOP symbol, 11.9% of products (n = 436) would indicate ‘high in’ sugar content, 13.2% of products (n = 487) would indicate ‘high in’ saturated fat content, and 19.4% of products (n = 712) would indicate ‘high in’ sodium content. The top 3 TRA categories with products that would display ‘high in’ sugar content were Desserts (65.7%; n = 46), Sugars & Sweets (52.0%; n = 53), and Beverages (44.4%; n = 36). The top 3 TRA categories with products that would display ‘high in’ saturated fat content were Dessert Toppings & Fillings (62.5%; n = 5), Dairy Products & Substitutes (46.4%; n = 97), and Combination Dishes (42.1%; n = 8). The top 3 TRA categories with products that would display ‘high in’ sodium content were Soups (92.8%; n = 180), Combination Dishes (89.5%; n = 17), and Sauces & Dips (54.9%; n = 39).

### FLIP 2017

**Fig. 2:**
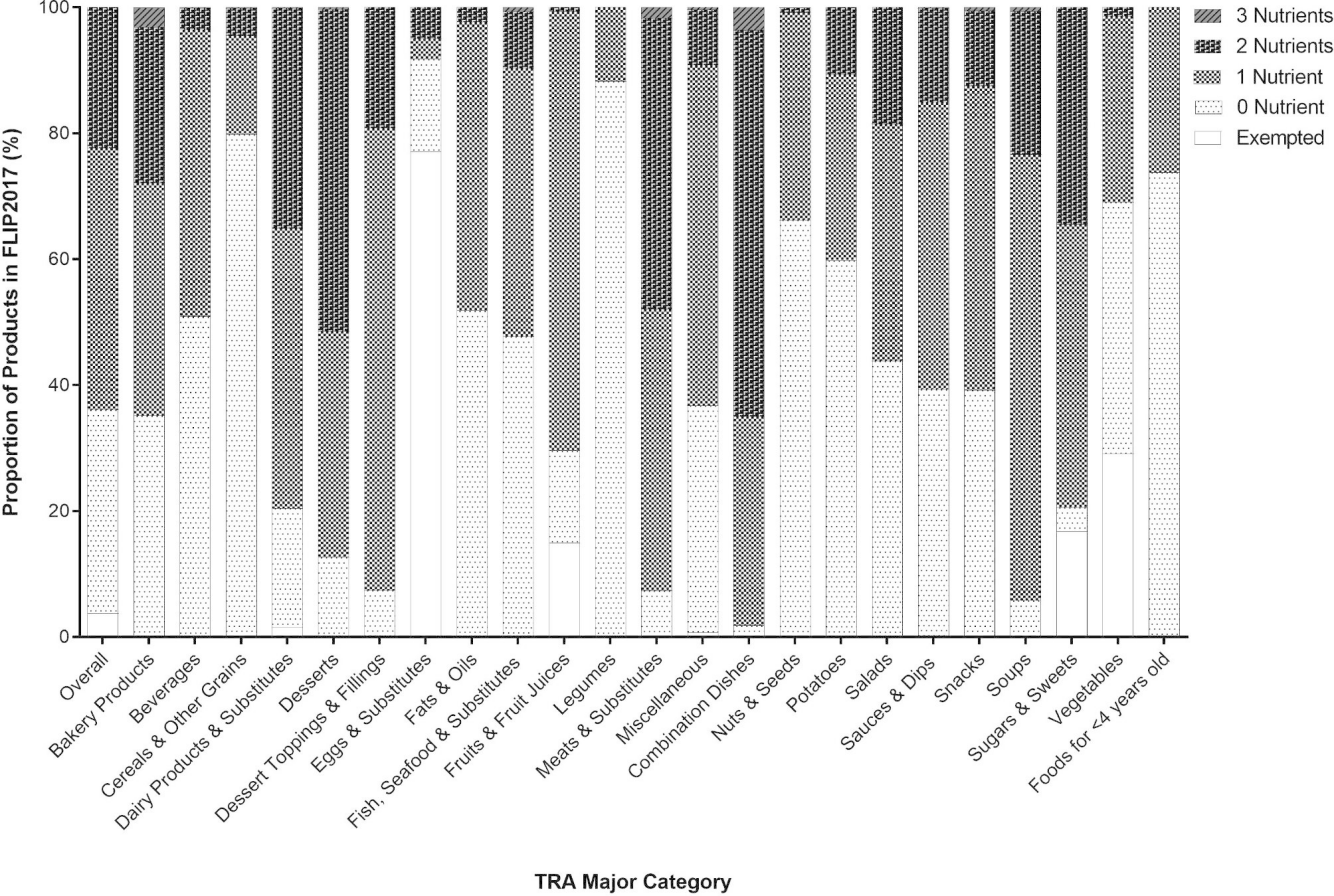
Proportion of food & beverage products in the Food Label Information Program (FLIP) 2017 that would be affected by the proposed Front-of-Pack (FOP) Labelling Regulations in Canada by Table of Reference Amounts (TRA) major food category. n = 17,521. A total of 13 (0.1%), 299 (1.7%), and 11 (0.1%) products were missing values for total sugars, saturated fats, and sodium, respectively, and were removed from analyses. Food and beverage products were divided into 5 categories: Exempted (i.e., exempted from FOP labelling regulations), 0 Nutrient (i.e., would not display a FOP symbol for having nutrient levels below the threshold levels), and 1–3 Nutrients (i.e., would display a FOP symbol for meeting or exceeding threshold levels for 1–3 nutrient(s) of concern)

Overall, 36.1% of products (n = 6,328) in FLIP 2017 would not be required to display a FOP symbol, while 63.9% of products (n = 11,193) would be required to display a FOP symbol. Among products that would not be required to display a FOP symbol, 10.5% of products (n = 666/6,328) would meet the exemption criteria of the proposed FOP labelling regulations and 89.5% of products (n = 5,662/6,328) would not exceed the any of the nutrient thresholds. Among products that would be required to display a FOP symbol, 65.2% (n = 7,296/11,193) would indicate one ‘high in’ nutrient, 33.3% (n = 3,733/11,193) would indicate two ‘high in’ nutrients, and 1.5% (n = 164/11,193) would indicate all three ‘high in’ nutrients. Figure 2 and **Supplementary Tables 5–6** show the proportion of products in FLIP 2017 categorized by the number of ‘high in’ nutrients indicated by a FOP symbol that products would display, presented by TRA major and minor food categories.

The top 3 TRA categories with products that would be exempted from the FOP labelling regulations were Eggs & Substitutes (77.0%, n = 47), Vegetables (29.2%, n = 254), and Sugars & Sweets (16.8%; n = 178). The top 3 TRA categories with products that would not display a FOP symbol, as nutrient levels would be below the thresholds were Legumes (88.3%; n = 166), Cereals & Other Grain Products (79.8%; n = 1,018), and Nuts & Seeds (66.3%; n = 169). The top 3 TRA categories with products that would display a FOP symbol for one ‘high in’ nutrient were Dessert Toppings & Fillings (73.4%; n = 69), Soups (70.8%; n = 340), and Fruits & Fruit Juices (69.7%; n = 740). The top 3 TRA categories with products that would display a FOP symbol for two ‘high in’ nutrients were Combination Dishes (61.3%; n = 699), Desserts (51.1%; n = 347), and Meats & Substitutes (46.3%, n = 445). The top 3 TRA categories with products that would display a FOP symbol for three ‘high in’ nutrient were Combination Dishes (3.6%; n = 41), Bakery Products (3.2%, n = 88), and Meats & Substitutes (1.7%, n = 16).

**Supplementary Table 7** shows the proportion of products in FLIP 2017 that would display a FOP symbol for each individual nutrient, by TRA major and minor categories. Overall, 26.9% of products (n = 4,709) would display a FOP symbol to indicate ‘high in’ sugar content, 28.6% (n = 5,018) would display a FOP symbol to indicate ‘high in’ saturated fat content, and 31.5% (n = 5,527) would display a FOP symbol to indicate ‘high in’ sodium content. The top 3 TRA categories that would display a FOP symbol to indicate ‘high in’ sugar content were Dessert Toppings & Fillings (92.6%, n = 87), Desserts (81.1%, n = 551), and Fruits & Fruit Juices (69.2%, n = 734). The top 3 TRA categories that would display a FOP symbol to indicate ‘high in’ ‘saturated fat’ content were Dairy Products & Substitutes (60.6%, n = 908), Combination Dishes (60.2%, n = 686), and Desserts (56.0%, n = 380). The top 3 TRA categories that would display a FOP symbol to indicate ‘high in’ sodium content were Combination Dishes (96.1%, n = 1,095), Soups (94.0%, n = 451), and Meats & Substitutes (83.6%, n = 804).

## Discussion

The objective of the study was to examine the prevalence of food and beverage products in Canada that would be impacted by the proposed Canadian FOP labelling regulations using the generic and the branded food composition databases. Overall, 35.4% of generic food products in CNF 2015 and 63.9% of branded packaged food products from FLIP 2017 would be required to display a FOP symbol. Among generic products, most products in Cereals & Other Grains, Legumes, and Nuts & Seeds categories would not display a FOP symbol, while most products in Soups, Combination Dishes, and Dessert Toppings & Fillings categories would display a FOP symbol. Among branded packaged products, most products in Eggs & Egg Substitutes, Legumes, Cereals & Other Grains, and Nuts & Seeds categories would not display a FOP symbol, while most products in Combination Dishes, Soups, Meats & Substitutes, and Desserts categories would display a FOP symbol. Both food databases revealed a FOP symbol indicating ‘high in’ sodium content would be the most prevalent FOP nutrient type on food and beverage products in Canada. Our findings show the potential for the proposed FOP labelling regulations to identify many packaged food and beverage products that are ‘high in’ nutrients-of-concern.

The results of the present study provide further evidence of the poor nutritional quality of the Canadian packaged food supply, supporting the need for national policies to improve the current retail food environment. Although the generic food composition database (i.e., CNF 2015) showed a lower prevalence of a FOP symbol among foods commonly consumed by Canadians, the branded database (i.e., FLIP 2017), showed that over 60% of packaged products would be deemed “less healthy” according to the proposed FOP labelling regulations (i.e., ‘high in’ one or more nutrients-of-concern). Consistent with our findings, much evidence has shown that the Canadian packaged food supply is dominated by energy-dense and nutrient-poor food and beverage products that are often highly processed [25–28]. Increased consumption of highly processed foods may be linked to poor diet quality and adverse health outcomes [30, 31], in part, related to the elevated amounts of nutrients-of-concern. As processed foods are more likely to undergo the addition of nutrients-of-concern, recommendations targeting processed foods are emerging globally. Suggestions to avoid highly processed foods have also been incorporated into the national dietary guidance documents of several countries, including Canada [32] and Brazil [33]. Nutrient profile models which underpin many nutrition policies and interventions have also focused on processing, for example, the Pan American Health Organization nutrient profiling model to identify the healthfulness of foods includes classifying foods based on the processing levels in addition to the levels of nutrients-of-concern [34]. Accounting for the differences between the CNF and FLIP with the former containing both packaged and unprocessed foods and beverages compared to the latter, which is mainly composed of packaged foods and beverages, the proposed FOP labelling regulations show the potential to identify packaged foods that are ‘high in’ nutrients-of-concern.

While products that would a FOP symbol were most prevalent overall in the packaged food supply, the number and type of ‘high in’ nutrients displayed varied at the category level, revealing that FOP labelling regulations will likely have category-specific effects in reducing the availability of nutrients-of-concern in the Canadian food supply. Similar FOP labelling regulations in Chile using mandatory nutrient-specific ‘high-in’ warning labels have been shown to promote product reformulations by manufacturers to decrease the availability of nutrients-of-concern in the food supply [7], which in turn can decrease the consumption of target nutrients. In about a year following the policy implementation in Chile, there was an overall decrease in the proportion of products displaying FOP labelling (51% vs. 44%) with category-specific decreases of target nutrients (e.g., beverages and breakfast cereals for sugars; cheeses and soups for sodium; and savoury spreads for saturated fats) [7]. Although the Chilean food supply reduced the number of products that displayed sugar and sodium FOP label in 5 and 6 categories (out of 16), respectively, only one food category reduced the number of products that displayed saturated fats [7]. Considering the target nutrients-of-concern play various roles in the processing of food and beverage products (e.g., controlling water activity, extending shelf-life, palatability) [35], some categories may take longer to reformulate, compared with other categories. As FOP labelling regulations are finalized and implemented in Canada, it will be vital to continue to monitor their impact on the food supply and make appropriate modifications to the regulations, if needed, for the health of Canadians.

Similar to our findings, food and beverage products that would display a FOP have been identified as key contributors of nutrients-of-concern. A previous study identified mixed dishes as one of the top 5 contributors of sodium and saturated fat intakes among Canadians [36], similar to our analysis showing the Combination Dishes category (e.g., pizzas, burritos, lasagna) with a high prevalence of products that would display a FOP symbol, particularly for saturated fat and/or sodium. However, the top contributors of sugars included a combination of products that would be exempted from the FOP labelling regulations (e.g., fresh and frozen fruits, unflavoured milk) and would be assessed to display a FOP (e.g., soda, 100% fruit juices, and dairy desserts) [36]. The difference in food categories is largely related to the exemption criteria introduced in the FOP labelling regulations, designed to distinguish products that would be composed of naturally-occurring nutrients (e.g., unsweetened fruits, vegetables, and milk) from those with added nutrients (e.g., sweetened fruits, pickled vegetables, flavoured milks). Although our analyses suggest that the proposed FOP labelling regulations would sufficiently differentiate products with naturally-occurring nutrients from those with added nutrients, the potential impact of the regulations on nutrient intakes of Canadians needs to be examined.

The most prevalent type of ‘high in’ nutrient indicated by a FOP symbol that would be found in the Canadian food supply system was sodium, likely related to excess intake levels among Canadians. Among the three nutrients-of-concern, Canadians most excessively consume sodium with an average intake of about 2,760 mg/d [37] compared to the recommended levels of Chronic Disease Risk Reduction Intake of 2,300 mg/d [38] (while Canadians consume 13% of total energy from free sugars [39] vs. 10% of the WHO recommended levels [40]; and ~ 10% of total energy from saturated fat [41] vs. 10% of the WHO recommended levels [42]). Despite the sodium reduction targets for processed foods introduced in 2012 for voluntary achievement by 2016 [43], many food categories have not met the target levels [44]. In addition to the updated sodium reduction targets set in 2020, the proposed mandatory FOP labelling regulations are needed to reduce the availability of sodium, particularly among products that have failed to meet the voluntary reduction targets. Furthermore, ongoing monitoring of national nutrient intakes is needed to assess the effectiveness of both mandatory and voluntary food and nutrition policies.

Our analysis using generic and branded food composition databases demonstrate the potential effectiveness of the proposed FOP labelling regulations in targeting packaged food and beverage products. About a third of the products in CNF 2015 were exempted from evaluation, most of which were fresh produce and meals that would typically not display a Nutrition Facts table under the *Food and Drugs Regulations* in Canada [45], whereas only 3.8% of products in FLIP 2017 were exempted, most of which were foods that have been shown to have some health benefits or well-known sources of target nutrients (i.e., fresh vegetables, fruits and unflavoured milks). Although a similar proportion of products that would exceed thresholds were observed (70% in CNF 2015 vs. 64% in FLIP 2017) after the removal of exempted foods, the branded food composition database provides a greater specificity and more realistic indication of the availability of products required to accurately assess the food supply system. Considering the generic food composition database uses the average nutrient levels of similar products in the Canadian market, the analysis of products based on threshold levels is difficult to assess. Our findings highlight the importance of branded food composition databases as a superior tool for evaluating FOP labelling and other nutrition policies.

This is the first study to date that examined the potential impact of the proposed Canadian FOP labelling regulations using food composition databases. With increasing global support for FOP labelling regulations to improve population health outcomes [46, 47], our findings contribute to a body of evidence supporting FOP labelling regulations to help determine the healthfulness of foods as the proposed Canadian FOP labelling regulations show the potential to similarly identify the healthfulness of foods. However, there are some limitations to note. First, this study did not examine the nutritional quality of the foods that would display a FOP symbol. In particular, the FOP labelling regulations target total sugar content even though health concerns associated with sugar intakes are related to free and/or added sugars [40]. In fact, a previous study has shown that the use of a free sugar threshold was more robust in identifying foods ‘high in’ (i.e., ≥ 15% DV) free sugar than using total sugars (54% vs. 37% of packaged foods in Canada in 2013) [17]. Therefore, future studies quantifying the difference in nutrients between foods that would and would not display a FOP symbol are warranted. Second, the cross-sectional nature of this study design provides a baseline analysis prior to the regulatory implementation of the FOP labelling policy. To monitor the effectiveness of the FOP labelling regulations, future studies examining the changes in the availability of nutrients-of-concern will be needed as the proposed regulations are finalized and implemented. Third, as a cross-sectional nutritional database of food and beverage products available in the Summer of 2017 in Ontario, FLIP 2017 does not include seasonal and region-specific products. Data collection at different times of the year are needed to examine the nutritional content of products available throughout the year. Lastly, this study was limited in that it did not analyze sales-weighted data, which is considered the gold standard in assessing the availability of nutrient(s) in the food supply because it takes into consideration the number of products sold within each food category to reflect product consumption. Future studies using sales data are needed to examine the consumption of products that would and would not display a FOP symbol to assess the impact of the proposed FOP labelling regulations.

## Conclusion

Our findings show that the proposed FOP labelling regulations in Canada would identify over 60% of packaged foods with excess contents of nutrients-of-concern. Although the FOP labelling have been shown to improve population dietary intakes through manufacturer-driven reformulations and/or changes in consumer behaviours, these data can serve as a baseline to determine the potential impact of the proposed Canadian FOP labelling regulations once the final FOP labelling regulations are implemented. Considering the high prevalence of packaged foods that would be affected by the proposed FOP labelling regulations, the timely release of the final FOP labelling regulations is needed.

## Electronic supplementary material

Below is the link to the electronic supplementary material.

Supplementary Material 1

## Data Availability

The Canadian Nutrient File (CNF) dataset is available online through Health Canada website: https://www.canada.ca/en/health-canada/services/food-nutrition/healthy-eating/nutrient-data/canadian-nutrient-file-about-us.html. Food Label Information Program (FLIP) dataset is copyrighted property of the L’Abbe Lab at University of Toronto, Health Canada, and Dietitians of Canada.

## Abbreviations

CNF: Canadian Nutrient File
FLIP: Food Label Information Program
FOP: Front-of-pack
TRA: Table of Reference Amounts for Food
%DV: Percent Daily Value
