# Supplementary material for: Evaluating the potential implications of canadian front-of-pack labelling regulations in generic and branded food composition databases
Source: BMC Public Health. 2022 Oct 6;22:1866. doi: 10.1186/s12889-022-14269-4 (PMC9535871; doi:10.1186/s12889-022-14269-4)
Supplement: Supplementary file 1 — Supplementary Material 1 [file 12889_2022_14269_MOESM1_ESM.docx]

**Supplementary Tables**

Table of Contents

[Supplementary Table 1. Nutrient thresholds to determine displaying a FOP symbol according to the proposed front-of-pack labelling regulations 2](#_Toc114460951)

[Supplementary Table 2. Summary of the proportion of foods that would display a FOP symbol in CNF2015 based on the proposed front-of-pack labelling regulations by TRA major category 3](#_Toc114460952)

[Supplementary Table 3. Summary of the expected proportion of a FOP symbol in CNF2015 based on the proposed FOP labelling regulations 4](#_Toc114460953)

[Supplementary Table 4. Summary of the nutrient types that would be displayed in a FOP symbol in CNF2015 based on the proposed FOP labelling regulations 13](#_Toc114460954)

[Supplementary Table 5. Summary of the proportion of foods that would display a FOP symbol in FLIP2017 based on the proposed front-of-pack labelling regulations by TRA major category 19](#_Toc114460955)

[Supplementary Table 6. Summary of the expected proportion of a FOP symbol in FLIP2017 based on the proposed FOP labelling regulations 20](#_Toc114460956)

[Supplementary Table 7. Summary of the nutrient types that would be displayed in a FOP symbol in FLIP2017 based on the proposed FOP labelling regulations 29](#_Toc114460957)

[References 35](#_Toc114460958)

# **Supplementary Table 1.** Nutrient thresholds to determine displaying a FOP symbol according to the proposed FOP labelling regulations

|  | **Nutrient-of-public health concern**^*^ | | |
| --- | --- | --- | --- |
|  | **Total Sugars** | **Saturated Fat** | **Sodium** |
| Foods or beverages | 15% DV (15 g) | 15% DV (3 g) | 15% DV (350 mg) |
| Meals and main dishes | 30% DV (30 g) | 30% DV (6 g) | 15% DV (690 mg) |
| Foods intended solely for 1- to 4-year-old children | 15% DV (8 g) | 15% DV (1.5 g) | 15% DV (230 mg) |

^*^The details of the proposed front-of-pack labelling regulations released in *Canada Gazette I [1]* included thresholds for nutrients-of-public health concern (i.e., total sugars, saturated fat, sodium) for a food or beverage product that would be assessed to display ‘high-in’ symbol(s. Abbreviations: %DV, percent daily value.

# Supplementary Table 2. Summary of the proportion of foods that would display a FOP symbol in CNF2015 based on the proposed FOP labelling regulations by TRA major category

|  |  | **FOP symbol based on the proposed FOP labelling regulations** | | | | | | |
| --- | --- | --- | --- | --- | --- | --- | --- | --- |
|  |  | **No FOP Symbol** | | | **FOP Symbol** | | | |
| **TRA Category^*^** | **n** | **Exempted** | **0 Nutrients** | **Total** | **1 Nutrient** | **2 Nutrients** | **3 Nutrients** | **Total** |
| A. Bakery Products | 254 | 0 | 106 (41.7%) | 106 (41.7%) | 119 (46.9%) | 29 (11.4%) | 0 | 148 (58.3%) |
| B. Beverages | 81 | 0 | 42 (51.9%) | 42 (51.9%) | 29 (35.8%) | 3 (3.7%) | 7 (8.6%) | 39 (48.1%) |
| C. Cereals & Other Grains | 188 | 0 | 172 (91.5%) | 172 (91.5%) | 16 (8.5%) | 0 | 0 | 16 (8.5%) |
| D. Dairy Products & Substitutes | 209 | 13 (6.2%) | 41 (19.6%) | 54 (25.8%) | 94 (45.0%) | 59 (28.2%) | 2 (1.0%) | 155 (74.2%) |
| E. Desserts | 70 | 0 | 11 (15.7%) | 11 (15.7%) | 39 (55.7%) | 20 (28.6%) | 0 | 59 (84.3%) |
| F. Dessert Toppings & Fillings | 8 | 0 | 1 (12.5%) | 1 (12.5%) | 6 (75.0%) | 1 (12.5%) | 0 | 7 (87.5%) |
| G. Eggs & Substitutes | 21 | 14 (66.7%) | 4 (19.0%) | 18 (85.7%) | 1 (4.8%) | 1 (4.8%) | 1 (4.8%) | 3 (14.3%) |
| H. Fats & Oils | 144 | 0 | 78 (54.2%) | 78 (54.2%) | 64 (44.4%) | 2 (1.4%) | 0 | 66 (45.8%) |
| I. Seafood & Substitutes | 251 | 96 (38.2%) | 106 (42.2%) | 202 (80.5%) | 46 (18.3%) | 3 (1.2%) | 0 | 49 (19.5%) |
| J. Fruits & Fruit Juices | 343 | 148 (43.1%) | 69 (20.1%) | 217 (63.3%) | 126 (36.7%) | 0 | 0 | 126 (36.7%) |
| K. Legumes | 119 | 0 | 113 (95.0%) | 113 (95.0%) | 6 (5.0%) | 0 | 0 | 6 (5.0%) |
| L. Meats & Substitutes | 863 | 600 (69.5%) | 49 (5.7%) | 649 (75.2%) | 133 (15.4%) | 81 (9.4%) | 0 | 214 (24.8%) |
| M. Miscellaneous | 80 | 1 (1.3%) | 66 (82.5%) | 67 (83.8%) | 10 (12.5%) | 3 (3.8%) | 0 | 13 (16.3%) |
| N. Combination Dishes | 19 | 0 | 2 (10.5%) | 2 (10.5%) | 9 (47.4%) | 8 (42.1%) | 0 | 17 (89.5%) |
| O. Nuts & Seeds | 95 | 0 | 86 (90.5%) | 86 (90.5%) | 9 (9.5%) | 0 | 0 | 9 (9.5%) |
| P. Potatoes | 27 | 14 (51.9%) | 8 (29.6%) | 22 (81.5%) | 4 (14.8%) | 1 (3.7%) | 0 | 5 (18.5%) |
| R. Sauces & Dips | 71 | 0 | 28 (39.4%) | 28 (39.4%) | 35 (49.3%) | 8 (11.3%) | 0 | 43 (60.6%) |
| S. Snacks | 54 | 0 | 29 (53.7%) | 29 (53.7%) | 17 (31.5%) | 8 (14.8%) | 0 | 25 (46.3%) |
| T. Soups | 194 | 0 | 11 (5.7%) | 11 (5.7%) | 141 (72.7%) | 37 (19.1%) | 5 (2.6%) | 183 (94.3%) |
| U. Sugars & Sweets | 102 | 35 (34.3%) | 10 (9.8%) | 45 (44.1%) | 31 (30.4%) | 26 (25.5%) | (0.0%) | 57 (55.9%) |
| V. Vegetables | 385 | 326 (84.7%) | 39 (10.1%) | 365 (94.8%) | 20 (5.2%) | 0 | 0 | 20 (5.2%) |
| W. Foods for <4 years old | 99 | 0 | 53 (53.5%) | 53 (53.5%) | 37 (37.4%) | 9 (9.1%) | 0 | 46 (46.5%) |
| **OVERALL TOTAL** | 3,677 | 1,247 (33.9%) | 1,124 (30.6%) | 2,371 (64.5%) | 992 (27.0%) | 299 (8.1%) | 15 (0.4%) | 1,306 (35.5%) |

All values are presented as n (%); n=3,677. ^*^TRA major categories were defined as per Health Canada’s Table of Reference Amounts for Foods [2]. A total of 354 (9.6%), 62 (1.7%), and 20 (0.5%) products were missing values for total sugars, saturated fats, and sodium, respectively. Abbreviations: CNF, Canadian Nutrient File; FOP, front-of-pack; TRA, Table of Reference Amounts for Foods.

# **Supplementary Table 3.** Summary of the expected proportion of a FOP symbol in CNF2015 based on the proposed FOP labelling regulations

|  |  | **FOP symbol based on the proposed FOP labelling regulations** | | | | | | | |
| --- | --- | --- | --- | --- | --- | --- | --- | --- | --- |
|  |  | **No FOP Symbol** | | | **FOP Symbol** | | | |  |
| **TRA Category^*^** | **n** | **Exempted** | **0 Nutrients** | **Total** | **1 Nutrient** | **2 Nutrients** | **3 Nutrients** | **Total** |  |
| **A. Bakery Products** | | | | | | | | | |
| A1. Bread, excluding sweet quick-type^†^ | 46 | 0 | 20 (43.5%) | 20 (43.5%) | 24 (52.2%) | 2 (4.3%) | 0 | 26 (56.5%) |  |
| A2. Tea biscuits, scones, rolls, buns, etc.^†^ | 32 | 0 | 24 (75.0%) | 24 (75.0%) | 6 (18.8%) | 2 (6.3%) | 0 | 8 (25.0%) |  |
| A3. Bagels, naan, flat bread^†^ | 14 | 0 | 3 (21.4%) | 3 (21.4%) | 9 (64.3%) | 2 (14.3%) | 0 | 11 (78.6%) |  |
| A4. Brownies ^†^ | 3 | 0 | 1 (33.3%) | 1 (33.3%) | 2 (66.7%) | 0 | 0 | 2 (66.7%) |  |
| A5. Heavy weight cake^†^ | 1 | 0 | 0 | 0 0 | 0 | 1 (100.0%) | 0 | 1 (100.0%) |  |
| A6. Medium weight cake^†^ | 12 | 0 | 0 | 0 0 | 2 (16.7%) | 10 (83.3%) | 0 | 12 (100.0%) |  |
| A7. Light weight cake | 1 | 0 | 0 | 0 0 | 0 | 1 (100.0%) | 0 | 1 (100.0%) |  |
| A8. coffee cakes, doughnuts, sweet quick-type breads, etc.^†^ | 12 | 0 | 3 (25.0%) | 3 (25.0%) | 9 (75.0%) | 0 | 0 | 9 (75.0%) |  |
| A9. Muffins | 1 | 0 | 0 | 0 0 | 0 | 1 (100.0%) | 0 | 1 (100.0%) |  |
| A10. Cookies^†^ | 46 | 0 | 19 (41.3%) | 19 (41.3%) | 19 (41.3%) | 8 (17.4%) | 0 | 27 (58.7%) |  |
| A11. Accompaniment crackers^†^ | 10 | 0 | 3 (30.0%) | 3 (30.0%) | 7 (70.0%) | 0 | 0 | 7 (70.0%) |  |
| A12. Snack crackers^†^ | 28 | 0 | 10 (35.7%) | 10 (35.7%) | 18 (64.3%) | 0 | 0 | 18 (64.3%) |  |
| A13. Dry breads^†^ | 6 | 0 | 5 (83.3%) | 5 (83.3%) | 1 (16.7%) | 0 | 0 | 1 (16.7%) |  |
| A14. Toaster pastries | 1 | 0 | 0 | 0 0 | 1 (100.0%) | 0 | 0 | 1 (100.0%) |  |
| A15. Ice cream cones | 2 | 0 | 2 (100.0%) | 2 (100.0%) | 0 | 0 | 0 | 0 0 |  |
| A16. Croutons^†^ | 2 | 0 | 2 (100.0%) | 2 (100.0%) | 0 | 0 | 0 | 0 0 |  |
| A17. French toast, pancakes, waffles^†^ | 9 | 0 | 0 | 0 0 | 9 (100.0%) | 0 | 0 | 9 (100.0%) |  |
| A18. Grain-based bars with filling | 4 | 0 | 0 | 0 0 | 3 (75.0%) | 1 (25.0%) | 0 | 4 (100.0%) |  |
| A19. Grain-based bars without filling^§^ | 11 | 0 | 8 (72.7%) | 8 (72.7%) | 2 (18.2%) | 1 (9.1%) | 0 | 3 (27.3%) |  |
| A20. Energy and protein bars | 5 | 0 | 1 (20.0%) | 1 (20.0%) | 4 (80.0%) | 0 | 0 | 4 (80.0%) |  |
| A21. Rice and corn cakes | 3 | 0 | 3 (100.0%) | 3 (100.0%) | 0 | 0 | 0 | 0 0 |  |
| A22. Pies, pastries, etc. | 1 | 0 | 0 | 0 0 | 1 (100.0%) | 0 | 0 | 1 (100.0%) |  |
| A23. Pie crust^†^ | 3 | 0 | 2 (66.7%) | 2 (66.7%) | 1 (33.3%) | 0 | 0 | 1 (33.3%) |  |
| A25. Taco shell | 1 | 0 | 0 | 0 0 | 1 (100.0%) | 0 | 0 | 1 (100.0%) |  |
| Category A Total | **254** | **0** | **106 (41.7%)** | **106 (41.7%)** | **119 (46.9%)** | **29 (11.4%)** | **0** | **148 (58.3%)** |  |
| **B. Beverages** | | | | | | | | | |
| B1. Carbonated and non-carbonated beverages^†^ | 45 | 0 | 23 (51.1%) | 23 (51.1%) | 21 (46.7%) | 1 (2.2%) | 0 | 22 (48.9%) |  |
| B3. Coffee^†^ | 19 | 0 | 13 (68.4%) | 13 (68.4%) | 2 (10.5%) | 1 (5.3%) | 3 (15.8%) | 6 (31.6%) |  |
| B4. Tea | 10 | 0 | 6 (60.0%) | 6 (60.0%) | 4 (40.0%) | 0 | 0 | 4 (40.0%) |  |
| B5. Cocoa and hot chocolate beverages | 7 | 0 | 0 | 0 0 | 2 (28.6%) | 1 (14.3%) | 4 (57.1%) | 7 (100.0%) |  |
| Category B Total | **81** | **0** | **42 (51.9%)** | **42 (51.9%)** | **29 (35.8%)** | **3 (3.7%)** | **7 (8.6%)** | **39 (48.1%)** |  |
| **C. Cereals & Other Grains** | | | | | | | | | |
| C1. Hot breakfast cereals^†,‡^ | 28 | 0 | 27 (96.4%) | 27 (96.4%) | 1 (3.6%) | 0 | 0 | 1 (3.6%) |  |
| C2. Ready-to-eat cereals, puffed and coated | 2 | 0 | 2 (100.0%) | 2 (100.0%) | 0 | 0 | 0 | 0 0 |  |
| C3. Ready-to-eat cereals, fruit and nut type | 16 | 0 | 9 (56.3%) | 9 (56.3%) | 7 (43.8%) | 0 | 0 | 7 (43.8%) |  |
| C4. Ready-to-eat cereals, granola type | 12 | 0 | 10 (83.3%) | 10 (83.3%) | 2 (16.7%) | 0 | 0 | 2 (16.7%) |  |
| C5. Bran and wheat germ, milled flax, etc.^†^ | 4 | 0 | 4 (100.0%) | 4 (100.0%) | 0 | 0 | 0 | 0 0 |  |
| C6. Flours and cornmeal^†^ | 28 | 0 | 27 (96.4%) | 27 (96.4%) | 1 (3.6%) | 0 | 0 | 1 (3.6%) |  |
| C7. Grains^†,‡^ | 58 | 0 | 57 (98.3%) | 57 (98.3%) | 1 (1.7%) | 0 | 0 | 1 (1.7%) |  |
| C8. Pastas^†^ | 23 | 0 | 22 (95.7%) | 22 (95.7%) | 1 (4.3%) | 0 | 0 | 1 (4.3%) |  |
| C9. Pastas (ready-to-eat)^†^ | 15 | 0 | 13 (86.7%) | 13 (86.7%) | 2 (13.3%) | 0 | 0 | 2 (13.3%) |  |
| C10. Starch | 1 | 0 | 1 (100.0%) | 1 (100.0%) | 0 | 0 | 0 | 0 0 |  |
| C11. Stuffing | 1 | 0 | 0 | 0 0 | 1 (100.0%) | 0 | 0 | 1 (100.0%) |  |
| **Category C Total** | **188** | 0 | 172 (91.5%) | 172 (91.5%) | 16 (8.5%) | 0 | 0 | 16 (8.5%) |  |
| **D. Dairy Products & Substitutes** | | | | | | | | | |
| D1. Cheese^†,‡,§^ | 60 | 0 | 4 (6.7%) | 4 (6.7%) | 31 (51.7%) | 25 (41.7%) | 0 | 56 (93.3%) |  |
| D2. Cottage cheese | 8 | 0 | 0 | 0 0 | 7 (87.5%) | 1 (12.5%) | 0 | 8 (100.0%) |  |
| D4. Hard cheese | 5 | 0 | 0 | 0 0 | 1 (20.0%) | 4 (80.0%) | 0 | 5 (100.0%) |  |
| D5. Quark | 9 | 0 | 0 | 0 0 | 3 (33.3%) | 6 (66.7%) | 0 | 9 (100.0%) |  |
| D6. Cream and cream substitutes^†^ | 10 | 0 | 6 (60.0%) | 6 (60.0%) | 3 (30.0%) | 1 (10.0%) | 0 | 4 (40.0%) |  |
| D7. Powder cream and cream substitutes | 3 | 0 | 3 (100.0%) | 3 (100.0%) | 0 | 0 | 0 | 0 0 |  |
| D8. Aerosol/whipped cream and cream substitutes | 4 | 0 | 0 | 0 0 | 4 (100.0%) | 0 | 0 | 4 (100.0%) |  |
| D9. Eggnog | 1 | 0 | 0 | 0 0 | 1 (100.0%) | 0 | 0 | 1 (100.0%) |  |
| D10. Evaporated/condensed milk | 4 | 0 | 3 (75.0%) | 3 (75.0%) | 1 (25.0%) | 0 | 0 | 1 (25.0%) |  |
| D11. Milk, buttermilk, milk-based drinks, plant-based milk substitutes^†^ | 41 | 13 (31.7%) | 8 (19.5%) | 21 (51.2%) | 10 (24.4%) | 8 (19.5%) | 2 (4.9%) | 20 (48.8%) |  |
| D13. Shakes | 7 | 0 | 1 (14.3%) | 1 (14.3%) | 3 (42.9%) | 3 (42.9%) | 0 | 6 (85.7%) |  |
| D14. Sour cream | 4 | 0 | 2 (50.0%) | 2 (50.0%) | 2 (50.0%) | 0 | 0 | 2 (50.0%) |  |
| D15. Yogurt | 53 | 0 | 14 (26.4%) | 14 (26.4%) | 28 (52.8%) | 11 (20.8%) | 0 | 39 (73.6%) |  |
| **Category D Total** | **209** | 13 (6.2%) | 41 (19.6%) | 54 (25.8%) | 94 (45.0%) | 59 (28.2%) | 2 (1.0%) | 155 (74.2%) |  |
| **E. Desserts** | | | | | | | | | |
| E1. Ice cream, frozen yogurt, sherbet, etc. in tubs | 24 | 0 | 1 (4.2%) | 1 (4.2%) | 11 (45.8%) | 12 (50.0%) | 0 | 23 (95.8%) |  |
| E2 Ice cream, frozen yogurt, sherbet, etc. as cakes, cones ^†^ | 9 | 0 | 1 (11.1%) | 1 (11.1%) | 6 (66.7%) | 2 (22.2%) | 0 | 8 (88.9%) |  |
| E3 Ice cream, frozen yogurt, sherbet, etc. as pops, bars ^†,‡^ | 10 | 0 | 5 (50.0%) | 5 (50.0%) | 5 (50.0%) | 0 | 0 | 5 (50.0%) |  |
| E4. Sundaes | 1 | 0 | 0 | 0 | 0 | 1 (100.0%) | 0 | 1 (100.0%) |  |
| E5. Custard, gelatin, pudding^†^ | 26 | 0 | 4 (15.4%) | 4 (15.4%) | 17 (65.4%) | 5 (19.2%) | 0 | 22 (84.6%) |  |
| **Category E Total** | **70** | **0** | **11 (15.7%)** | **11 (15.7%)** | **39 (55.7%)** | **20 (28.6%)** | **0** | **59 (84.3%)** |  |
| **F. Dessert Toppings & Fillings** | | | | | | | | | |
| F1. Dessert toppings | 6 | 0 | 1 (16.7%) | 1 (16.7%) | 4 (66.7%) | 1 (16.7%) | 0 | 5 (83.3%) |  |
| F2. Cake frostings | 2 | 0 | 0 | 0 | 2 (100.0%) | 0 | 0 | 2 (100.0%) |  |
| **Category F Total** | **8** | 0 | 1 (12.5%) | 1 (12.5%) | 6 (75.0%) | 1 (12.5%) | 0 | 7 (87.5%) |  |
| **G. Eggs & Substitutes** | | | | | | | | | |
| G1. Egg mixtures | 1 | 0 | 1 (100.0%) | 1 (100.0%) | 0 | 0 | 0 | 0 |  |
| G2. Eggs | 16 | 14 (87.5%) | 0 | 14 (87.5%) | 1 (6.3%) | 1 (6.3%) | 0 | 2 (12.5%) |  |
| G3. Egg substitutes | 4 | 0 | 3 (75.0%) | 3 (75.0%) | 0 | 0 | 1 (25.0%) | 1 (25.0%) |  |
| **Category G Total** | **21** | **14 (66.7%)** | **4 (19.0%)** | **18 (85.7%)** | **1 (4.8%)** | **1 (4.8%)** | **1 (4.8%)** | **3 (14.3%)** |  |
| **H. Fats & Oils** | | | | | | | | | |
| H1. Butter, margarine, lard, etc.^†,‡,§^ | 60 | 0 | 40 (66.7%) | 40 (66.7%) | 20 (33.3%) | 0 | 0 | 20 (33.3%) |  |
| H2. Vegetable oil^†^ | 33 | 0 | 28 (84.8%) | 28 (84.8%) | 5 (15.2%) | 0 | 0 | 5 (15.2%) |  |
| H3. Butter replacement | 1 | 0 | 1 (100.0%) | 1 (100.0%) | 0 | 0 | 0 | 0 0 |  |
| H4. Dressings for salad | 39 | 0 | 4 (10.3%) | 4 (10.3%) | 33 (84.6%) | 2 (5.1%) | 0 | 35 (89.7%) |  |
| H5. Mayonnaise and mayonnaise-type dressing | 10 | 0 | 4 (40.0%) | 4 (40.0%) | 6 (60.0%) | 0 | 0 | 6 (60.0%) |  |
| H6. Spray oil | 1 | 0 | 1 (100.0%) | 1 (100.0%) | 0 | 0 | 0 | 0 0 |  |
| **Category H Total** | **144** | **0** | **78 (54.2%)** | **78 (54.2%)** | **64 (44.4%)** | **2 (1.4%)** | **0** | **66 (45.8%)** |  |
| **I.** **Seafood & Substitutes** | | | | | | | | | |
| I1. Anchovies, caviar | 2 | 0 | 2 (100.0%) | 2 (100.0%) | 0 | 0 | 0 | 0 0 |  |
| I2. Marine and freshwater animals with sauce^†^ | 4 | 0 | 2 (50.0%) | 2 (50.0%) | 2 (50.0%) | 0 | 0 | 2 (50.0%) |  |
| I3. Marine and freshwater animals without sauce ^†,‡,§^ | 201 | 96 (47.8%) | 76 (37.8%) | 172 (85.6%) | 27 (13.4%) | 2 (1.0%) | 0 | 29 (14.4%) |  |
| I4. Canned marine and freshwater animals | 18 | 0 | 16 (88.9%) | 16 (88.9%) | 2 (11.1%) | 0 | 0 | 2 (11.1%) |  |
| I5. Smoked/pickled marine and freshwater animals | 26 | 0 | 10 (38.5%) | 10 (38.5%) | 15 (57.7%) | 1 (3.8%) | 0 | 16 (61.5%) |  |
| **Category I Total** | **251** | **96 (38.2%)** | **106 (42.2%)** | **202 (80.5%)** | **46 (18.3%)** | **3 (1.2%)** | **0** | **49 (19.5%)** |  |
| **J. Fruits & Fruit Juices** | | | | | | | | | |
| J1. Fruits (fresh, frozen, canned, coated, and uncoated)^†,‡^ | 127 | 77 (60.6%) | 16 (12.6%) | 93 (73.2%) | 34 (26.8%) | 0 | 0 | 34 (26.8%) |  |
| J2. Berries^†^ | 48 | 32 (66.7%) | 7 (14.6%) | 39 (81.3%) | 9 (18.8%) | 0 | 0 | 9 (18.8%) |  |
| J3. Melons | 6 | 6 (100.0%) | 0 | 6 (100.0%) | 0 | 0 | 0 | 0 0 |  |
| J4. Avocados | 1 | 1 (100.0%) | 0 | 1 (100.0%) | 0 | 0 | 0 | 0 0 |  |
| J6. Apple sauces | 3 | 1 (33.3%) | 1 (33.3%) | 2 (66.7%) | 1 (33.3%) | 0 | 0 | 1 (33.3%) |  |
| J7. Dried fruits^†^ | 37 | 26 (70.3%) | 8 (21.6%) | 34 (91.9%) | 3 (8.1%) | 0 | 0 | 3 (8.1%) |  |
| J8. Candied/pickled fruits^†,‡^ | 10 | 0 | 8 (80.0%) | 8 (80.0%) | 2 (20.0%) | 0 | 0 | 2 (20.0%) |  |
| J9. Fruits for garnish | 5 | 5 (100.0%) | 0 | 5 (100.0%) | 0 | 0 | 0 | 0 0 |  |
| J10. Fruit relishes^†^ | 2 | 0 | 2 (100.0%) | 2 (100.0%) | 0 | 0 | 0 | 0 0 |  |
| J11. Juices, nectars, fruit drinks^†,‡^ | 99 | 0 | 22 (22.2%) | 22 (22.2%) | 77 (77.8%) | 0 | 0 | 77 (77.8%) |  |
| J12. Fruit juices used as ingredients | 5 | 0 | 5 (100.0%) | 5 (100.0%) | 0 | 0 | 0 | 0 0 |  |
| **Category J Total** | **343** | 148 (43.1%) | 69 (20.1%) | 217 (63.3%) | 126 (36.7%) | 0 | 0 | 126 (36.7%) |  |
| **K. Legumes** | | | | | | | | | |
| K1. Tofu or tempeh^†^ | 5 | 0 | 5 (100.0%) | 5 (100.0%) | 0 | 0 | 0 | 0 |  |
| K2. Beans, lentils, etc.^†^ | 114 | 0 | 108 (94.7%) | 108 (94.7%) | 6 (5.3%) | 0 | 0 | 6 (5.3%) |  |
| **Category K Total** | **119** | **0** | **113 (95.0%)** | **113 (95.0%)** | **6 (5.0%)** | **0** | **0** | **6 (5.0%)** |  |
| **L. Meats & Substitutes** | | | | | | | | | |
| L1. Pork rinds and bacon^‡^ | 5 | 0 | 1 (20.0%) | 1 (20.0%) | 1 (20.0%) | 3 (60.0%) | 0 | 4 (80.0%) |  |
| L2. Beef, pork and poultry breakfast strips | 5 | 0 | 0 | 0 0 | 1 (20.0%) | 4 (80.0%) | 0 | 5 (100.0%) |  |
| L3. Dried meat and poultry^†,‡^ | 15 | 0 | 6 (40.0%) | 6 (40.0%) | 2 (13.3%) | 7 (46.7%) | 0 | 9 (60.0%) |  |
| L4. Luncheon meats | 44 | 0 | 2 (4.5%) | 2 (4.5%) | 25 (56.8%) | 17 (38.6%) | 0 | 42 (95.5%) |  |
| L5. Sausage products^†^ | 67 | 0 | 5 (7.5%) | 5 (7.5%) | 26 (38.8%) | 36 (53.7%) | 0 | 62 (92.5%) |  |
| L6. Cust of meat & poultry without sauce^†^ | 666 | 600 (90.1%) | 27 (4.1%) | 627 (94.1%) | 33 (5.0%) | 6 (0.9%) | 0 | 39 (5.9%) |  |
| L7. Patties, ground meat with and without breading | 28 | 0 | 6 (21.4%) | 6 (21.4%) | 21 (75.0%) | 1 (3.6%) | 0 | 22 (78.6%) |  |
| L8. Cured meats^‡^ | 16 | 0 | 1 (6.3%) | 1 (6.3%) | 13 (81.3%) | 2 (12.5%) | 0 | 15 (93.8%) |  |
| L9. Canned meats^†^ | 16 | 0 | 1 (6.3%) | 1 (6.3%) | 11 (68.8%) | 4 (25.0%) | 0 | 15 (93.8%) |  |
| L10. Meat and poultry with sauce | 1 | 0 | 0 | 0 0 | 0 | 1 (100.0%) | 0 | 1 (100.0%) |  |
| **Category L Total** | **863** | **600 (69.5%)** | **49 (5.7%)** | **649 (75.2%)** | **133 (15.4%)** | **81 (9.4%)** | **0** | **214 (24.8%)** |  |
| **M. Miscellaneous** | | | | | | | | | |
| M1. Baking powder, baking soda, yeast | 10 | 0 | 9 (90.0%) | 9 (90.0%) | 1 (10.0%) | 0 | 0 | 1 (10.0%) |  |
| M3. Bread crumbs^†,‡^ | 6 | 0 | 1 (16.7%) | 1 (16.7%) | 5 (83.3%) | 0 | 0 | 5 (83.3%) |  |
| M5. Cocoa powder | 2 | 0 | 2 (100.0%) | 2 (100.0%) | 0 | 0 | 0 | 0 0 |  |
| M7. Chewing gum | 2 | 0 | 2 (100.0%) | 2 (100.0%) | 0 | 0 | 0 | 0 0 |  |
| M9. Salt, salt substitutes | 3 | 1 (33.3%) | 2 (66.7%) | 3 (100.0%) | 0 | 0 | 0 | 0 0 |  |
| M10. Spices and herbs without salt^†^ | 50 | 0 | 50 (100.0%) | 50 (100.0%) | 0 | 0 | 0 | 0 0 |  |
| M11. Coconut milk^†^ | 4 | 0 | 0 | 0 0 | 3 (75.0%) | 1 (25.0%) | 0 | 4 (100.0%) |  |
| M12. Dried coconut | 3 | 0 | 0 | 0 0 | 1 (33.3%) | 2 (66.7%) | 0 | 3 (100.0%) |  |
| **Category M Total** | **80** | **1 (1.3%)** | **66 (82.5%)** | **67 (83.8%)** | **10 (12.5%)** | **3 (3.8%)** | **0** | **13 (16.3%)** |  |
| **N. Combination Dishes** | | | | | | | | | |
| N1. Combination dishes^†^ | 15 | 0 | 2 (13.3%) | 2 (13.3%) | 8 (53.3%) | 5 (33.3%) | 0 | 13 (86.7%) |  |
| N2. Burritos, pizzas, sandwiches, meat pie, etc. | 3 | 0 | 0 | 0 | 0 | 3 (100.0%) | 0 | 3 (100.0%) |  |
| N3. Hors d’oeuvres | 1 | 0 | 0 | 0 | 1 (100.0%) | 0 | 0 | 1 (100.0%) |  |
| **Category N Total** | **19** | **0** | **2 (10.5%)** | **2 (10.5%)** | **9 (47.4%)** | **8 (42.1%)** | **0** | **17 (89.5%)** |  |
| **O. Nuts & Seeds** | | | | | | | | | |
| O1. Nuts and seeds (not used for snacks)^†^ | 69 | 0 | 61 (88.4%) | 61 (88.4%) | 8 (11.6%) | 0 | 0 | 8 (11.6%) |  |
| O2. Nut pastes and creams | 1 | 0 | 0 | 0 | 1 (100.0%) | 0 | 0 | 1 (100.0%) |  |
| O3. Nut butters^†^ | 12 | 0 | 12 (100.0%) | 12 (100.0%) | 0 | 0 | 0 | 0 |  |
| O4. Nut flours^†^ | 13 | 0 | 13 (100.0%) | 13 (100.0%) | 0 | 0 | 0 | 0 |  |
| **Category O Total** | **95** | **0** | **86 (90.5%)** | **86 (90.5%)** | **9 (9.5%)** | **0** | **0** | **9 (9.5%)** |  |
| **P. Potatoes** | | | | | | | | | |
| P1. French fries^†^ | 5 | 0 | 4 (80.0%) | 4 (80.0%) | 1 (20.0%) | 0 | 0 | 1 (20.0%) |  |
| P2. Mashed, stuffed, candied potatoes^†^ | 4 | 0 | 1 (25.0%) | 1 (25.0%) | 2 (50.0%) | 1 (25.0%) | 0 | 3 (75.0%) |  |
| P3. Fresh, canned, frozen potatoes^†,‡^ | 18 | 14 (77.8%) | 3 (16.7%) | 17 (94.4%) | 1 (5.6%) | 0 | 0 | 1 (5.6%) |  |
| **Category P Total** | **27** | **14 (51.9%)** | **8 (29.6%)** | **22 (81.5%)** | **4 (14.8%)** | **1 (3.7%)** | **0** | **5 (18.5%)** |  |
| **R. Sauces & Dips** | | | | | | | | | |
| R1. Dipping sauces^†^ | 5 | 0 | 3 (60.0%) | 3 (60.0%) | 2 (40.0%) | 0 | 0 | 2 (40.0%) |  |
| R2. Dips and spreads^†^ | 3 | 0 | 2 (66.7%) | 2 (66.7%) | 1 (33.3%) | 0 | 0 | 1 (33.3%) |  |
| R3. Major main entrée sauce^†^ | 9 | 0 | 1 (11.1%) | 1 (11.1%) | 4 (44.4%) | 4 (44.4%) | 0 | 8 (88.9%) |  |
| R4. Minor main entrée sauce^†^ | 27 | 0 | 7 (25.9%) | 7 (25.9%) | 16 (59.3%) | 4 (14.8%) | 0 | 20 (74.1%) |  |
| R5. Major condiments | 12 | 0 | 5 (41.7%) | 5 (41.7%) | 7 (58.3%) | 0 | 0 | 7 (58.3%) |  |
| R6. Minor condiments^†,‡^ | 15 | 0 | 10 (66.7%) | 10 (66.7%) | 5 (33.3%) | 0 | 0 | 5 (33.3%) |  |
| **Category R Total** | **71** | **0** | **28 (39.4%)** | **28 (39.4%)** | **35 (49.3%)** | **8 (11.3%)** | **0** | **43 (60.6%)** |  |
| **S. Snacks** | | | | | | | | | |
| S1. Chips, pretzels, etc.^†^ | 45 | 0 | 25 (55.6%) | 25 (55.6%) | 14 (31.1%) | 6 (13.3%) | 0 | 20 (44.4%) |  |
| S2. Nuts or seeds (used as snacks)^†^ | 7 | 0 | 4 (57.1%) | 4 (57.1%) | 3 (42.9%) | 0 | 0 | 3 (42.9%) |  |
| S3. Meat/poultry snack food sticks | 2 | 0 | 0 | 0 0 | 0 | 2 (100.0%) | 0 | 2 (100.0%) |  |
| **Category S Total** | **54** | **0** | **29 (53.7%)** | **29 (53.7%)** | **17 (31.5%)** | **8 (14.8%)** | **0** | **25 (46.3%)** |  |
| **T. Soups** | | | | | | | | | |
| T1. All varieties of soups (includes broth)^†^ | 194 | 0 | 11 (5.7%) | 11 (5.7%) | 141 (72.7%) | 37 (19.1%) | 5 (2.6%) | 183 (94.3%) |  |
| **Category T Total** | **194** | **0** | **11 (5.7%)** | **11 (5.7%)** | **141 (72.7%)** | **37 (19.1%)** | **5 (2.6%)** | **183 (94.3%)** |  |
| **U. Sugars & Sweets** | | | | | | | | | |
| U1. Candies, confectionaries, chocolates^†^ | 40 | 0 | 0 | 0 0 | 18 (45.0%) | 22 (55.0%) | 0 | 40 (100.0%) |  |
| U2. After dinner confectionaries | 1 | 0 | 0 | 0 0 | 1 (100.0%) | 0 | 0 | 1 (100.0%) |  |
| U3. Hard candies^†^ | 4 | 0 | 2 (50.0%) | 2 (50.0%) | 2 (50.0%) | 0 | 0 | 2 (50.0%) |  |
| U4. Baking candies | 5 | 0 | 0 | 0 0 | 1 (20.0%) | 4 (80.0%) | 0 | 5 (100.0%) |  |
| U5. Breath mints | 1 | 0 | 1 (100.0%) | 1 (100.0%) | 0 | 0 | 0 | 0 0 |  |
| U6. Roll-type candies | 3 | 0 | 3 (100.0%) | 3 (100.0%) | 0 | 0 | 0 | 0 0 |  |
| U7. Icing sugar | 5 | 5 (100.0%) | 0 | 5 (100.0%) | 0 | 0 | 0 | 0 0 |  |
| U8. Honey, molasses, bread spreads | 6 | 3 (50.0%) | 2 (33.3%) | 5 (83.3%) | 1 (16.7%) | 0 | 0 | 1 (16.7%) |  |
| U9. Jams, jellies, fruit spreads | 6 | 0 | 0 | 0 0 | 6 (100.0%) | 0 | 0 | 6 (100.0%) |  |
| U10. Fruit leather^†^ | 3 | 0 | 2 (66.7%) | 2 (66.7%) | 1 (33.3%) | 0 | 0 | 1 (33.3%) |  |
| U11. Marshmallows | 1 | 0 | 0 | 0 0 | 1 (100.0%) | 0 | 0 | 1 (100.0%) |  |
| U12. Sugars | 3 | 3 (100.0%) | 0 | 3 (100.0%) | 0 | 0 | 0 | 0 0 |  |
| U14. Syrups used as toppings | 17 | 17 (100.0%) | 0 | 17 (100.0%) | 0 | 0 | 0 | 0 0 |  |
| U15. Syrups used as ingredients | 6 | 6 (100.0%) | 0 | 6 (100.0%) | 0 | 0 | 0 | 0 0 |  |
| **Category U Total** | **102** | **35 (34.3%)** | **10 (9.8%)** | **45 (44.1%)** | **31 (30.4%)** | **26 (25.5%)** | **(0.0%)** | **57 (55.9%)** |  |
| **V. Vegetables** | | | | | | | | | |
| V1. Vegetables without sauce^†^ | 315 | 291 (92.4%) | 20 (6.3%) | 311 (98.7%) | 4 (1.3%) | 0 | 0 | 4 (1.3%) |  |
| V3. Vegetables used for garnishing/flavouring | 1 | 0 | 0 | 0 0 | 1 (100.0%) | 0 | 0 | 1 (100.0%) |  |
| V4. Chili pepper & green onion | 5 | 5 (100.0%) | 0 | 5 (100.0%) | 0 | 0 | 0 | 0 0 |  |
| V5. Seaweed, dehydrated mushrooms^†^ | 14 | 13 (92.9%) | 0 | 13 (92.9%) | 1 (7.1%) | 0 | 0 | 1 (7.1%) |  |
| V6. Sprouts^†^ | 14 | 13 (92.9%) | 0 | 13 (92.9%) | 1 (7.1%) | 0 | 0 | 1 (7.1%) |  |
| V7. Vegetable juice and drink | 1 | 1 (100.0%) | 0 | 1 (100.0%) | 0 | 0 | 0 | 0 0 |  |
| V8. Olives | 6 | 1 (16.7%) | 2 (33.3%) | 3 (50.0%) | 3 (50.0%) | 0 | 0 | 3 (50.0%) |  |
| V9. Sun-dried tomatoes and other pickled or oil-packed vegetables^†^ | 3 | 0 | 2 (66.7%) | 2 (66.7%) | 1 (33.3%) | 0 | 0 | 1 (33.3%) |  |
| V10. Relish | 20 | 0 | 13 (65.0%) | 13 (65.0%) | 7 (35.0%) | 0 | 0 | 7 (35.0%) |  |
| V11. Vegetable paste | 1 | 0 | 0 | 0 0 | 1 (100.0%) | 0 | 0 | 1 (100.0%) |  |
| V12. Vegetable sauce or purée | 2 | 1 (50.0%) | 0 | 1 (50.0%) | 1 (50.0%) | 0 | 0 | 1 (50.0%) |  |
| **Category V Total** | **385** | **326 (84.7%)** | **39 (10.1%)** | **365 (94.8%)** | **20 (5.2%)** | **0** | **0** | **20 (5.2%)** |  |
| **W.** **Foods for <4 years old** | | | | | | | | | |
| W1. Cereals to be prepared | 34 | 0 | 18 (52.9%) | 18 (52.9%) | 11 (32.4%) | 5 (14.7%) | 0 | 16 (47.1%) |  |
| W2. Ready-to-eat cereals and cereal bars | 3 | 0 | 3 (100.0%) | 3 (100.0%) | 0 | 0 | 0 | 0 0 |  |
| W3. Cookies, biscuits, etc. | 8 | 0 | 8 (100.0%) | 8 (100.0%) | 0 | 0 | 0 | 0 0 |  |
| W4. Strained meat, desserts, combination dishes | 44 | 0 | 23 (52.3%) | 23 (52.3%) | 19 (43.2%) | 2 (4.5%) | 0 | 21 (47.7%) |  |
| W5. Combination dishes | 6 | 0 | 1 (16.7%) | 1 (16.7%) | 4 (66.7%) | 1 (16.7%) | 0 | 5 (83.3%) |  |
| W6. Juices | 3 | 0 | 0 | 0 0 | 3 (100.0%) | 0 | 0 | 3 (100.0%) |  |
| W7. Yogurt | 1 | 0 | 0 | 0 0 | 0 | 1 (100.0%) | 0 | 1 (100.0%) |  |
| **Category W Total** | **99** | **0** | **53 (53.5%)** | **53 (53.5%)** | **37 (37.4%)** | **9 (9.1%)** | **0** | **46 (46.5%)** |  |
| **OVERALL TOTAL** | **3,677** | **1,247 (33.9%)** | **1,124 (30.6%)** | **2,371 (64.5%)** | **992 (27.0%)** | **299 (8.1%)** | **15 (0.4%)** | **1,306 (35.5%)** |  |

All values are presented as n (%); n=3,677. *Major and minor categories were defined as per Health Canada’s Table of Reference Amounts for Foods[2]. ^†^Indicates categories with products that were missing values for total sugars (n=354; 9.6% overall). ^‡^Indicates categories with products that were missing values for saturated fats (n=62; 1.7% overall). ^§^Indicates categories with products that were missing values for sodium (n=20; 0.5% overall). Abbreviations: CNF, Canadian Nutrient File; FOP, front-of-pack; TRA, Table of Reference Amounts for Foods.

# **Supplementary Table 4.** Summary of the nutrient types that would be displayed in a FOP symbol in CNF 2015 based on the proposed FOP labelling regulations

|  |  | **FOP Symbol “High in” Nutrient Type** | | |
| --- | --- | --- | --- | --- |
| **TRA Category^*^** | **n** | **Total Sugars** | **Saturated Fat** | **Sodium** |
| **A. Bakery Products** | | | | |
| A1. Bread, excluding sweet quick-type | 46 | 0^†^ | 26 (56.5%) | 2 (4.3%) |
| A2. Tea biscuits, scones, rolls, buns, etc. | 32 | 0^†^ | 8 (25.0%) | 2 (6.3%) |
| A3. Bagels, naan, flat bread | 14 | 0^†^ | 10 (71.4%) | 3 (21.4%) |
| A4. Brownies | 3 | 2 (66.7%)^†^ | 0 | 0 |
| A5. Heavy weight cake | 1 | 0^†^ | 1 (100.0%) | 1 (100.0%) |
| A6. Medium weight cake | 12 | 9 (75.0%)^†^ | 12 (100.0%) | 1 (8.3%) |
| A7. Light weight cake | 1 | 1 (100.0%) | 1 (100.0%) | 0 |
| A8. coffee cakes, doughnuts, sweet quick-type breads, etc. | 12 | 3 (25.0%)^†^ | 0 | 6 (50.0%) |
| A9. Muffins | 1 | 1 (100.0%) | 1 (100.0%) | 0 |
| A10. Cookies | 46 | 22 (47.8%)^†^ | 0 | 13 (28.3%) |
| A11. Accompaniment crackers | 10 | 0^†^ | 7 (70.0%) | 0 |
| A12. Snack crackers | 28 | 0^†^ | 17 (60.7%) | 1 (3.6%) |
| A13. Dry breads | 6 | 0^†^ | 0 | 1 (16.7%) |
| A14. Toaster pastries | 1 | 1 (100.0%) | 0 | 0 |
| A15. Ice cream cones | 2 | 0 | 0 | 0 |
| A16. Croutons | 2 | 0^†^ | 0 | 0 |
| A17. French toast, pancakes, waffles | 9 | 0^†^ | 9 (100.0%) | 0 |
| A18. Grain-based bars with filling | 4 | 4 (100.0%) | 0 | 1 (25.0%) |
| A19. Grain-based bars without filling | 11 | 2 (18.2%) | 0 | 2 (18.2%)^†^ |
| A20. Energy and protein bars | 5 | 3 (60.0%) | 1 (20.0%) | 0 |
| A21. Rice and corn cakes | 3 | 0 | 0 | 0 |
| A22. Pies, tarts, pastries, etc. | 1 | 0 | 1 (100.0%) | 0 |
| A23. Pie crust^†^ | 3 | 0^†^ | 1 (33.3%) | 0 |
| A25. Taco shell | 1 | 0 | 0 | 1 (100.0%) |
| **Category A Total** | **254** | **48 (18.9%)** | **34 (13.4%)** | **95 (37.4%)** |
| **B. Beverages** | | | | |
| B1. Carbonated and non-carbonated beverages | 45 | 22 (48.9%)^†^ | 0 | 1 (2.2%)^†^ |
| B3. Coffee | 19 | 4 (21.1%)^†^ | 5 (26.3%) | 4 (21.1%) |
| B4. Tea | 10 | 4 (40.0%) | 0 | 0 |
| B5. Cocoa and hot chocolate beverages | 7 | 6 (85.7%) | 6 (85.7%) | 4 (57.1%) |
| **Category B Total** | **81** | **36 (44.4%)** | **11 (13.6%)** | **9 (11.1%)** |
| **C. Cereals & Other Grains** | | | | |
| C1. Hot breakfast cereals | 28 | 0^†^ | 0^†^ | 1 (3.6%)^†^ |
| C2. Ready-to-eat cereals, puffed and coated | 2 | 0 | 0 | 0 |
| C3. Ready-to-eat cereals, fruit and nut type | 16 | 6 (37.5%)^†^ | 0 | 1 (6.3%) |
| C4. Ready-to-eat cereals, granola type | 12 | 0 | 2 (16.7%) | 0 |
| C5. Bran and wheat germ, milled flax, etc. | 4 | 0^†^ | 0 | 0 |
| C6. Flours and cornmeal | 28 | 1 (3.6%)^†^ | 0 | 0 |
| C7. Grains | 58 | 0^†^ | 0^†^ | 1 (1.7%) |
| C8. Pastas | 23 | 0^†^ | 0 | 1 (4.3%) |
| C9. Pastas (ready-to-eat) | 15 | 0^†^ | 0 | 2 (13.3%) |
| C10. Starch | 1 | 0 | 0 | 0 |
| C11. Stuffing | 1 | 0 | 0 | 1 (100.0%) |
| **Category C Total** | **188** | **7 (3.7%)** | **2 (1.1%)** | **7 (3.7%)** |
| **D. Dairy Products & Substitutes** | | | | |
| D1. Cheese | 60 | 0^†^ | 47 (78.3%)^†^ | 34 (56.7%)^†^ |
| D2. Cottage cheese | 8 | 0 | 1 (12.5%) | 8 (100.0%) |
| D4. Hard cheese | 5 | 0 | 5 (100.0%) | 4 (80.0%) |
| D5. Quark | 9 | 0 | 8 (88.9%) | 7 (77.8%) |
| D6. Cream and cream substitutes | 10 | 1 (11.1%)^†^ | 4 (40.0%) | 0 |
| D7. Powder cream and cream substitutes | 3 | 0 | 0 | 0 |
| D8. Aerosol/whipped cream and cream substitutes | 4 | 0 | 4 (100.0%) | 0 |
| D9. Eggnog | 1 | 0 | 1 (100.0%) | 0 |
| D10. Evaporated/condensed milk | 4 | 1 (25.0%) | 0 | 0 |
| D11. Milk, buttermilk, milk-based drinks, plant-based milk substitutes | 41 | 13 (36.1%)^†^ | 8 (19.5%) | 10 (24.4%) |
| D13. Shakes | 7 | 3 (42.9%) | 2 (28.6%) | 4 (57.1%) |
| D14. Sour cream | 4 | 0 | 2 (50.0%) | 0 |
| D15. Yogurt | 53 | 35 (66.0%) | 15 (28.3%) | 0 |
| **Category D Total** | **209** | **54 (25.8%)** | **97 (46.4%)** | **67 (32.1%)** |
| **E. Desserts** | | | | |
| E1. Ice cream, frozen yogurt, sherbet, etc. in tubs | 24 | 19 (79.2%) | 16 (66.7%) | 0 |
| E2 Ice cream, frozen yogurt, sherbet, etc. as cakes, cones | 9 | 6 (66.7%)^†^ | 4 (44.4%) | 0 |
| E3 Ice cream, frozen yogurt, sherbet, etc. as pops, bars | 10 | 4 (40.0%)^†^ | 1 (10.0%)^†^ | 0 |
| E4. Sundaes | 1 | 1 (100.0%) | 1 (100.0%) | 0 |
| E5. Custard, gelatin, pudding | 26 | 16 (61.5%)^†^ | 0 | 11 (42.3%) |
| **Category E Total** | **70** | **46 (65.7%)** | **22 (31.4%)** | **11 (15.7%)** |
| **F. Dessert Toppings & Fillings** | | | | |
| F1. Dessert toppings | 6 | 1 (16.7%) | 5 (83.3%) | 0 |
| F2. Cake frostings | 2 | 2 (100.0%) | 0 | 0 |
| **Category F Total** | **8** | **3 (37.5%)** | **5 (62.5%)** | **0** |
| **G. Eggs & Substitutes** | | | | |
| G1. Egg mixtures | 1 | 0 | 0 | 0 |
| G2. Eggs | 16 | 0 | 2 (12.5%) | 1 (6.3%) |
| G3. Egg substitutes | 4 | 1 (25.0%) | 1 (25.0%) | 1 (25.0%) |
| **Category G Total** | **21** | **1 (4.8%)** | **3 (14.3%)** | **2 (9.5%)** |
| **H. Fats & Oils** | | | | |
| H1. Butter, margarine, lard, etc. | 60 | 0^†^ | 20 (33.3%)^†^ | 0^†^ |
| H2. Vegetable oil | 33 | 0^†^ | 5 (15.2%) | 0 |
| H3. Butter replacement | 1 | 0 | 0 | 0 |
| H4. Dressings for salad | 39 | 1 (2.6%) | 1 (2.6%) | 35 (89.7%) |
| H5. Mayonnaise & mayonnaise-type dressings | 10 | 0^†^ | 0^†^ | 6 (60.0%) |
| H6. Spray oil | 1 | 0 | 0 | 0 |
| **Category H Total** | **144** | **1 (0.7%)** | **26 (18.1%)** | **41 (28.5%)** |
| **I. Fish, Seafood & Substitutes** | | | | |
| I1. Anchovies, caviar | 2 | 0 | 0 | 0^†^ |
| I2. Marine & freshwater animals with sauce^†^ | 4 | 0^†^ | 1 (25.0%) | 1 (25.0%) |
| I3. Marine & freshwater animals without sauce | 201 | 0^†^ | 10 (5.0%)^†^ | 21 (10.4%)^†^ |
| I4. Canned marine and freshwater animals | 18 | 0 | 0 | 2 (11.1%) |
| I5. Smoked/pickled marine & freshwater animals | 26 | 0 | 3 (11.5%)^†^ | 14 (53.8%) |
| **Category I Total** | **251** | **0** | **14 (5.6%)** | **38 (15.1%)** |
| **J. Fruits & Fruit Juices** | | | | |
| J1. Fruits (fresh, frozen, canned, coated, and uncoated) | 127 | 33 (26.0%)^†^ | 1 (0.8%)^†^ | 0 |
| J2. Berries | 48 | 9 (18.8%)^†^ | 0 | 0 |
| J3. Melons | 6 | 0 | 0 | 0 |
| J4. Avocados | 1 | 0 | 0 | 0 |
| J6. Apple sauces | 3 | 1 (33.3%) | 0 | 0 |
| J7. Dried fruits | 37 | 3 (8.1%)^†^ | 0 | 0 |
| J8. Candied/pickled fruits | 10 | 2 (20.0%)^†^ | 0^†^ | 0 |
| J9. Fruits for garnish | 5 | 0 | 0 | 0 |
| J10. Fruit relishes | 2 | 0^†^ | 0 | 0 |
| J11. Juices, nectars, fruit drinks | 99 | 77 (77.8%)^†^ | 0^†^ | 0 |
| J12. Fruit juices used as ingredients | 5 | 0 | 0 | 0 |
| **Category J Total** | **343** | **125 (36.4%)** | **1 (0.3%)** | **0** |
| **K. Legumes** | | | | |
| K1. Tofu or tempeh | 5 | 0^†^ | 0 | 0 |
| K2. Beans, lentils, peas, etc. | 114 | 0^†^ | 0 | 6 (5.3%) |
| **Category K Total** | **119** | **0** | **0** | **6 (5.0%)** |
| **L. Meats & Substitutes** | | | | |
| L1. Pork rinds and bacon | 5 | 0 | 3 (60.0%)^†^ | 4 (80.0%) |
| L2. Beef, pork and poultry breakfast strips | 5 | 0 | 4 (80.0%) | 5 (100.0%) |
| L3. Dried meat and poultry | 15 | 0^†^ | 7 (46.7%)^†^ | 9 (60.0%) |
| L4. Luncheon meats | 44 | 0 | 17 (38.6%) | 42 (95.5%) |
| L5. Sausage products | 67 | 0^†^ | 38 (56.7%) | 60 (89.6%) |
| L6. Cust of meat & poultry without sauce | 666 | 0^†^ | 36 (5.4%) | 9 (1.4%) |
| L7. Patties, ground meats with and without breading | 28 | 0 | 21 (75.0%) | 2 (7.1%) |
| L8. Cured meats | 16 | 0 | 2 (12.5%)^†^ | 15 (93.8%) |
| L9. Canned meats | 16 | 0^†^ | 5 (31.3%) | 14 (87.5%) |
| L10. Meat and poultry with sauce | 1 | 0 | 1 (100.0%) | 1 (100.0%) |
| **Category L Total** | **863** | **0** | **134 (15.5%)** | **161 (18.7%)** |
| **M. Miscellaneous** | | | | |
| M1. Baking powder, baking soda, yeast | 10 | 0^†^ | 0 | 1 (10.0%) |
| M3. Breadcrumbs | 6 | 0^†^ | 1 (16.7%)^†^ | 4 (66.7%) |
| M5. Cocoa powder | 2 | 0 | 0 | 0 |
| M7. Chewing gum | 2 | 0 | 0 | 0 |
| M9. Salt, salt substitutes | 3 | 0 | 0 | 0 |
| M10. Spices and herbs without salt | 50 | 0^†^ | 0 | 0 |
| M11. Coconut milk | 4 | 1 (25.0%)^†^ | 4 (100.0%) | 0 |
| M12. Dried coconut | 3 | 2 (66.7%) | 3 (100.0%) | 0 |
| **Category M Total** | **80** | **3 (3.8%)** | **8 (10.0%)** | **5 (6.3%)** |
| **N. Combination Dishes** | | | | |
| N1. Combination dishes | 15 | 0^†^ | 5 (33.3%) | 13 (86.7%) |
| N2. Burritos, pizzas, sandwiches, etc. | 3 | 0 | 3 (100.0%) | 3 (100.0%) |
| N3. Hors d’oeuvres | 1 | 0 | 0 | 1 (100.0%) |
| **Category N Total** | **19** | **0** | **8 (42.1%)** | **17 (89.5%)** |
| **O. Nuts & Seeds** | | | | |
| O1. Nuts & seeds (not used for snacks) | 69 | 1 (1.4%)^†^ | 7 (10.1%) | 0 |
| O2. Nut pastes and creams | 1 | 1 (100.0%) | 0 | 0 |
| O3. Nut butters | 12 | 0^†^ | 0 | 0 |
| O4. Nut flours | 13 | 0^†^ | 0 | 0 |
| **Category O Total** | **95** | **2 (2.1%)** | **7 (7.4%)** | **0** |
| **P. Potatoes** | | | | |
| P1. French fries | 5 | 0^†^ | 0 | 1 (20.0%) |
| P2. Mashed, stuffed, candied potatoes | 4 | 0^†^ | 2 (50.0%) | 2 (50.0%) |
| P3. Fresh, canned, frozen potatoes | 18 | 1 (5.6%)^†^ | 0 | 0 |
| **Category P Total** | **27** | **1 (3.7%)** | **2 (7.4%)** | **3 (11.1%)** |
| **R. Sauces & Dips** | | | | |
| R1. Dipping sauces | 5 | 1 (20.0%)^†^ | 0 | 1 (20.0%) |
| R2. Dips and spreads | 3 | 0^†^ | 0 | 1 (33.3%) |
| R3. Major main entrée sauce | 9 | 0^†^ | 4 (44.4%) | 8 (88.9%) |
| R4. Minor main entrée sauce | 27 | 1 (3.7%)^†^ | 6 (22.2%) | 17 (63.0%) |
| R5. Major condiments | 12 | 0 | 0 | 7 (58.3%) |
| R6. Minor condiments | 15 | 0^†^ | 0^†^ | 5 (33.3%) |
| **Category R Total** | **71** | **2 (2.8%)** | **10 (14.1%)** | **39 (54.9%)** |
| **S. Snacks** | | | | |
| S1. Chips, pretzels, etc. | 45 | 4 (8.9%)^†^ | 14 (31.1%) | 8 (17.8%) |
| S2. Nuts or seeds (used as snacks) | 7 | 0^†^ | 2 (28.6%) | 1 (14.3%) |
| S3. Meat/poultry snack food sticks | 2 | 0 | 2 (100.0%) | 2 (100.0%) |
| **Category S Total** | **54** | **4 (7.4%)** | **18 (33.3%)** | **11 (20.4%)** |
| **T. Soups** | | | | |
| T1. All varieties of soups (includes broth) | 194 | 11 (5.7%)^†^ | 39 (20.1%)^†^ | 180 (92.8%) |
| **Category T Total** | **194** | **11 (5.7%)** | **39 (20.1%)** | **180 (92.8%)** |
| **U. Sugars & Sweets** | | | | |
| U1. Candies, confectionaries, chocolates | 40 | 38 (95.0%)^†^ | 24 (60.0%) | 0 |
| U2. After dinner confectionaries | 1 | 1 (100.0%) | 0 | 0 |
| U3. Hard candies | 4 | 2 (50.0%)^†^ | 0 | 0 |
| U4. Baking candies | 5 | 4 (80.0%) | 5 (100.0%) | 0 |
| U5. Breath mints | 1 | 0 | 0 | 0 |
| U6. Roll-type candies | 3 | 0 | 0 | 0 |
| U7. Icing sugar | 5 | 0 | 0 | 0 |
| U8. Honey, molasses, bread spreads | 6 | 0 | 1 (16.7%) | 0 |
| U9. Jams, jellies, fruit spreads | 6 | 6 (100.0%) | 0 | 0 |
| U10. Fruit leather | 3 | 1 (33.3%)^†^ | 0 | 0 |
| U11. Marshmallows | 1 | 1 (100.0%) | 0 | 0 |
| U12. Sugars | 3 | 0 | 0 | 0 |
| U14. Syrups used as toppings | 17 | 0 | 0 | 0 |
| U15. Syrups used as ingredients | 6 | 0 | 0 | 0 |
| **Category U Total** | **102** | **53 (52.0%)** | **30 (29.4%)** | **0** |
| **V. Vegetables** | | | | |
| V1. Vegetables without sauce | 315 | 0^†^ | 0 | 4 (1.3%) |
| V3. Vegetables used for garnishing/flavouring | 1 | 0^†^ | 0 | 1 (100.0%) |
| V4. Chili pepper & green onion | 5 | 0 | 0 | 0 |
| V5. Seaweed, dehydrated mushrooms | 14 | 0^†^ | 0 | 1 (7.1%) |
| V6. Sprouts | 14 | 0 | 0 | 1 (7.1%) |
| V7. Vegetable juice and drink | 1 | 0 | 0 | 0 |
| V8. Olives | 6 | 0 | 0 | 3 (50.0%) |
| V9. Sun-dried tomatoes and other pickled or oil-packed vegetables | 3 | 0^†^ | 0 | 1 (33.3%) |
| V10. Relish | 20 | 1 (5.0%) | 0 | 6 (30.0%) |
| V11. Vegetable paste | 1 | 0 | 0 | 1 (100.0%) |
| V12. Vegetable sauce or purée | 2 | 0 | 0 | 1 (50.0%) |
| **Category V Total** | **385** | **1 (0.3%)** | **0** | **19 (4.9%)** |
| **W. Foods for <4 years old** | | | | |
| W1. Cereals to be prepared | 34 | 16 (47.1%) | 5 (14.7%) | 0 |
| W2. Ready-to-eat cereals and cereal bars | 3 | 0 | 0^†^ | 0 |
| W3. Cookies, biscuits, etc. | 8 | 0 | 0^†^ | 0 |
| W4. Strained meat, desserts, combination dishes | 44 | 17 (38.6%) | 5 (11.4%) | 1 (2.3%) |
| W5. Combination dishes | 6 | 1 (16.7%) | 5 (83.3%) | 0 |
| W6. Juices | 3 | 3 (100.0%) | 0 | 0 |
| W7. Yogurt | 1 | 1 (100.0%) | 1 (100.0%) | 0 |
| **Category W Total** | **99** | **38 (38.4%)** | **16 (16.2%)** | **1 (1.0%)** |
| **OVERALL TOTAL** | **3,677** | **436 (11.9%)** | **487 (13.2%)** | **712 (19.4%)** |

All values are presented as n (%); n=3,677. ^*^Major and minor categories were defined as per Health Canada’s Table of Reference Amounts for Foods[2].^†^Indicates categories with missing nutrient values. A total of 354 (9.6%), 62 (1.7%), and 20 (0.5%) products overall were missing values for total sugars, saturated fats, and sodium, respectively. Abbreviations: CNF, Canadian Nutrient File; FOP, front-of-pack; TRA, Table of Reference Amounts for Foods.

# **Supplementary** Table 5. Summary of the proportion of foods that would display a FOP symbol in FLIP2017 based on the proposed front-of-pack labelling regulations by TRA major category

|  |  | **FOP symbol based on the proposed FOP labelling regulations** | | | | | | | |
| --- | --- | --- | --- | --- | --- | --- | --- | --- | --- |
|  |  | **No FOP Symbol** | | | **FOP Symbol** | | | | |
| **TRA Category^*^** | **n** | **Exempted** | **1 Nutrient** | **Total** | **1 Nutrient** | **2 Nutrients** | **3 Nutrients** | **Total** |  |
| A. Bakery Products | 2,775 | 0 | 976 (35.2%) | 976 (35.2%) | 1,026 (37.0%) | 685 (24.7%) | 88 (3.2%) | 1,799 (64.8%) |  |
| B. Beverages | 851 | 0 | 433 (50.9%) | 433 (50.9%) | 388 (45.6%) | 29 (3.4%) | 1 (0.1%) | 418 (49.1%) |  |
| C. Cereals & Other Grains | 1,275 | 0 | 1,018 (79.8%) | 1,018 (79.8%) | 200 (15.7%) | 56 (4.4%) | 1 (0.1%) | 257 (20.2%) |  |
| D. Dairy Products & Substitutes | 1,498 | 24 (1.6%) | 283 (18.9%) | 307 (20.5%) | 666 (44.5%) | 524 (35.0%) | 1 (0.1%) | 1.191 (79.5%) |  |
| E. Desserts | 679 | 0 | 86 (12.7%) | 86 (12.7%) | 244 (35.9%) | 347 (51.1%) | 2 (0.3%) | 593 (87.3%) |  |
| F. Dessert Toppings & Fillings | 94 | 0 | 7 (7.4%) | 7 (7.4%) | 69 (73.4%) | 18 (19.1%) | 0 | 87 (92.6%) |  |
| G. Eggs & Substitutes | 61 | 47 (77.0%) | 9 (14.8%) | 56 (91.8%) | 2 (3.3%) | 2 (3.3%) | 0 | 4 (6.2%) |  |
| H. Fats & Oils | 656 | 0 | 340 (51.8%) | 340 (51.8%) | 301 (45.9%) | 15 (2.3%) | 0 | 316 (48.2%) |  |
| I. Seafood & Substitutes | 446 | 0 | 213 (47.8%) | 213 (47.8%) | 190 (42.6%) | 40 (9.0%) | 3 (0.7%) | 233 (52.2%) |  |
| J. Fruits & Fruit Juices | 1,061 | 159 (15.0%) | 155 (14.6%) | 314 (29.6%) | 740 (69.7%) | 7 (0.7%) | 0 | 747 (70.4%) |  |
| K. Legumes | 188 | 0 | 166 (88.3%) | 166 (88.3%) | 22 (11.7%) | 0 | 0 | 22 (11.7%) |  |
| L. Meats & Substitutes | 962 | 0 | 71 (7.4%) | 71 (7.4%) | 430 (44.7%) | 445 (46.3%) | 16 (1.7%) | 891 (92.6%) |  |
| M. Miscellaneous | 557 | 4 (0.7%) | 201 (36.1%) | 205 (36.8%) | 301 (54.0%) | 49 (8.8%) | 2 (0.4%) | 352 (63.2%) |  |
| N. Combination Dishes | 1,140 | 0 | 21 (1.8%) | 21 (1.8%) | 379 (33.2%) | 699 (61.3%) | 41 (3.6%) | 1119 (98.2%) |  |
| O. Nuts & Seeds | 255 | 0 | 169 (66.3%) | 169 (66.3%) | 84 (32.9%) | 2 (0.8%) | 0 | 86 (33.7%) |  |
| P. Potatoes | 132 | 0 | 79 (59.8%) | 79 (59.8%) | 39 (29.5%) | 14 (10.6%) | 0 | 53 (40.2%) |  |
| Q. Salads | 130 | 0 | 57 (43.8%) | 57 (43.8%) | 49 (37.7%) | 24 (18.5%) | 0 | 73 (56.2%) |  |
| R. Sauces & Dips | 1,250 | 0 | 492 (39.4%) | 492 (39.4%) | 570 (45.6%) | 187 (15.0%) | 1 (0.1%) | 758 (60.6%) |  |
| S. Snacks | 866 | 0 | 340 (39.3%) | 340 (39.3%) | 418 (48.3%) | 103 (11.9%) | 5 (0.6%) | 526 (60.7%) |  |
| T. Soups | 480 | 0 | 28 (5.8%) | 28 (5.8%) | 340 (70.8%) | 109 (22.7%) | 3 (0.6%) | 452 (94.2%) |  |
| U. Sugars & Sweets | 1,062 | 178 (16.8%) | 41 (3.9%) | 219 (20.6%) | 478 (45.0%) | 365 (34.4%) | 0 | 843 (79.4%) |  |
| V. Vegetables | 871 | 254 (29.2%) | 348 (40.0%) | 602 (69.1%) | 257 (29.5%) | 12 (1.4%) | 0 | 269 (30.9%) |  |
| W. Foods for <4 years old | 232 | 0 | 129 (55.6%) | 129 (55.6%) | 103 (44.4%) | 0 | 0 | 103 (44.4%) |  |
| **OVERALL TOTAL** | 17,521 | 666 (3.8%) | 5,662 (32.3%) | 6,328 (36.1%) | 7,296 (41.6%) | 3,733 (21.3%) | 164 (0.9%) | 11,193 (63.9%) |  |

All values are presented as n (%); n=17,521. *Major and minor categories were defined as per Health Canada’s Table of Reference Amounts for Foods [2]. A total of 13 (0.1%), 299 (1.7%), and 11 (0.1%) products were missing values for total sugars, saturated fats, and sodium, respectively.

# **Supplementary Table 6.** Summary of the expected proportion of a FOP symbol in FLIP2017 based on the proposed FOP labelling regulations

|  |  | **FOP symbol based on the proposed FOP labelling regulations** | | | | | | |
| --- | --- | --- | --- | --- | --- | --- | --- | --- |
|  |  | **No FOP Symbol** | | | **FOP Symbol** | | | |
| **TRA Category^*^** | **n** | **Exempted** | **0 Nutrient** | **Total** | **1 Nutrient** | **2 Nutrients** | **3 Nutrients** | **Total** |
| **A. Bakery Products** | | | | | | | | |
| A1. Bread, excluding sweet quick-type | 340 | 0 | 187 (55.0%) | 187 (55.0%) | 147 (43.2%) | 6 (1.8%) | 0 | 153 (45.0%) |
| A2. Tea biscuits, scones, rolls, buns, etc. | 290 | 0 | 171 (59.0%) | 171 (59.0%) | 102 (35.2%) | 17 (5.9%) | 0 | 119 (41.0%) |
| A3. Bagels, naan, flat bread | 86 | 0 | 25 (29.1%) | 25 (29.1%) | 58 (67.4%) | 3 (3.5%) | 0 | 61 (70.9%) |
| A4. Brownies | 29 | 0 | 2 (6.9%) | 2 (6.9%) | 15 (51.7%) | 12 (41.4%) | 0 | 27 (93.1%) |
| A5. Heavy weight cake | 68 | 0 | 0 | 0 | 0 | 48 (70.6%) | 20 (29.4%) | 68 (100.0%) |
| A6. Medium weight cake | 155 | 0 | 0 | 0 | 20 (12.9%) | 105 (67.7%) | 30 (19.4%) | 155 (100.0%) |
| A7. Light weight cake | 5 | 0 | 0 | 0 | 4 (80.0%) | 1 (20.0%) | 0 | 5 (100.0%) |
| A8. coffee cakes, doughnuts, sweet quick-type breads, etc. | 111 | 0 | 19 (17.1%) | 19 (17.1%) | 55 (49.5%) | 27 (24.3%) | 10 (9.0%) | 92 (82.9%) |
| A9. Muffins | 40 | 0 | 1 (2.5%) | 1 (2.5%) | 3 (7.5%) | 26 (65.0%) | 10 (25.0%) | 39 (97.5%) |
| A10. Cookies^‡^ | 523 | 0 | 98 (18.7%) | 98 (18.7%) | 163 (31.2%) | 262 (50.1%) | 0 | 425 (81.3%) |
| A11. Accompaniment crackers^‡^ | 254 | 0 | 145 (57.1%) | 145 (57.1%) | 92 (36.2%) | 17 (6.7%) | 0 | 109 (42.9%) |
| A12. Snack crackers | 66 | 0 | 12 (18.2%) | 12 (18.2%) | 50 (75.8%) | 4 (6.1%) | 0 | 54 (81.8%) |
| A13. Dry breads | 88 | 0 | 47 (53.4%) | 47 (53.4%) | 35 (39.8%) | 6 (6.8%) | 0 | 41 (46.6%) |
| A14. Toaster pastries | 11 | 0 | 5 (45.5%) | 5 (45.5%) | 6 (54.5%) | 0 | 0 | 6 (54.5%) |
| A15. Ice cream cones | 20 | 0 | 18 (90.0%) | 18 (90.0%) | 2 (10.0%) | 0 | 0 | 2 (10.0%) |
| A16. Croutons | 37 | 0 | 33 (89.2%) | 33 (89.2%) | 4 (10.8%) | 0 | 0 | 4 (10.8%) |
| A17. French toast, pancakes, waffles | 54 | 0 | 8 (14.8%) | 8 (14.8%) | 44 (81.5%) | 1 (1.9%) | 1 (1.9%) | 46 (85.2%) |
| A18. Grain-based bars with filling | 105 | 0 | 36 (34.3%) | 36 (34.3%) | 40 (38.1%) | 29 (27.6%) | 0 | 69 (65.7%) |
| A19. Grain-based bars without filling | 94 | 0 | 63 (67.0%) | 63 (67.0%) | 25 (26.6%) | 6 (6.4%) | 0 | 31 (33.0%) |
| A20. Energy and protein bars^‡^ | 205 | 0 | 74 (36.1%) | 74 (36.1%) | 101 (49.3%) | 29 (14.1%) | 1 (0.5%) | 131 (63.9%) |
| A21. Rice and corn cakes | 35 | 0 | 15 (42.9%) | 15 (42.9%) | 18 (51.4%) | 2 (5.7%) | 0 | 20 (57.1%) |
| A22. Pies, tarts, etc. | 114 | 0 | 0 | 0 | 16 (14.0%) | 82 (71.9%) | 16 (14.0%) | 114 (100.0%) |
| A23. Pie crust | 18 | 0 | 0 | 0 | 17 (94.4%) | 1 (5.6%) | 0 | 18 (100.0%) |
| A24. Pizza crust | 14 | 0 | 11 (78.6%) | 11 (78.6%) | 3 (21.4%) | 0 | 0 | 3 (21.4%) |
| A25. Taco shell | 13 | 0 | 6 (46.2%) | 6 (46.2%) | 6 (46.2%) | 1 (7.7%) | 0 | 7 (53.8%) |
| **Category A Total** | **2,775** | **0** | **976 (35.2%)** | **976 (35.2%)** | **1,026 (37.0%)** | **685 (24.7%)** | **88 (3.2%)** | **1,799 (64.8%)** |
| **B. Beverages** | | | | | | | | |
| B1. Carbonated and non-carbonated beverages^†,‡^ | 718 | 0 | 340 (47.4%) | 340 (47.4%) | 362 (50.4%) | 15 (2.1%) | 1 (0.1%) | 378 (52.6%) |
| B3. Coffee^†,‡,§^ | 28 | 0 | 21 (75.0%) | 21 (75.0%) | 6 (21.4%) | 1 (3.6%) | 0 | 7 (25.0%) |
| B4. Tea | 73 | 0 | 65 (89.0%) | 65 (89.0%) | 6 (8.2%) | 2 (2.7%) | 0 | 8 (11.0%) |
| B5. Cocoa and hot chocolate beverages | 32 | 0 | 7 (21.9%) | 7 (21.9%) | 14 (43.8%) | 11 (34.4%) | 0 | 25 (78.1%) |
| **Category B Total** | **851** | **0** | **433 (50.9%)** | **433 (50.9%)** | **388 (45.6%)** | **29 (3.4%)** | **1 (0.1%)** | **418 (49.1%)** |
| **C. Cereals & Other Grains** | | | | | | | | |
| C1. Hot breakfast cereals | 118 | 0 | 102 (86.4%) | 102 (86.4%) | 16 (13.6%) | 0 | 0 | 16 (13.6%) |
| C2. Ready-to-eat cereals, puffed and coated | 4 | 0 | 4 (100.0%) | 4 (100.0%) | 0 | 0 | 0 | 0 |
| C3. Ready-to-eat cereals, fruit and nut type | 88 | 0 | 49 (55.7%) | 49 (55.7%) | 37 (42.0%) | 2 (2.3%) | 0 | 39 (44.3%) |
| C4. Ready-to-eat cereals, granola type^‡^ | 169 | 0 | 130 (76.9%) | 130 (76.9%) | 35 (20.7%) | 4 (2.4%) | 0 | 39 (23.1%) |
| C5. Bran and wheat germ, milled flax, etc. | 38 | 0 | 37 (97.4%) | 37 (97.4%) | 1 (2.6%) | 0 | 0 | 1 (2.6%) |
| C6. Flours and cornmeal | 66 | 0 | 64 (97.0%) | 64 (97.0%) | 2 (3.0%) | 0 | 0 | 2 (3.0%) |
| C7. Grains | 278 | 0 | 197 (70.9%) | 197 (70.9%) | 80 (28.8%) | 1 (0.4%) | 0 | 81 (29.1%) |
| C8. Pastas | 493 | 0 | 424 (86.0%) | 424 (86.0%) | 23 (4.7%) | 45 (9.1%) | 1 (0.2%) | 69 (14.0%) |
| C10. Starch | 11 | 0 | 9 (81.8%) | 9 (81.8%) | 2 (18.2%) | 0 | 0 | 2 (18.2%) |
| C11. Stuffing | 10 | 0 | 2 (20.0%) | 2 (20.0%) | 4 (40.0%) | 4 (40.0%) | 0 | 8 (80.0%) |
| **Category C Total** | **1,275** | **0** | **1,018 (79.8%)** | **1,018 (79.8%)** | **200 (15.7%)** | **56 (4.4%)** | **1 (0.1%)** | **257 (20.2%)** |
| **D. Dairy Products & Substitutes** | | | | | | | | |
| D1. Cheese^‡^ | 569 | 0 | 2 (0.4%) | 2 (0.4%) | 268 (47.1%) | 299 (52.5%) | 0 | 567 (99.6%) |
| D2. Cottage cheese | 21 | 0 | 19 (90.5%) | 19 (90.5%) | 2 (9.5%) | 0 | 0 | 2 (9.5%) |
| D3. Cheese used as ingredients | 14 | 0 | 7 (50.0%) | 7 (50.0%) | 7 (50.0%) | 0 | 0 | 7 (50.0%) |
| D4. Hard cheese | 45 | 0 | 0 | 0 | 12 (26.7%) | 33 (73.3%) | 0 | 45 (100.0%) |
| D5. Quark | 110 | 0 | 4 (3.6%) | 4 (3.6%) | 24 (21.8%) | 82 (74.5%) | 0 | 106 (96.4%) |
| D6. Cream and cream substitutes | 41 | 0 | 10 (24.4%) | 10 (24.4%) | 25 (61.0%) | 6 (14.6%) | 0 | 31 (75.6%) |
| D7. Powder cream and cream substitute | 9 | 0 | 6 (66.7%) | 6 (66.7%) | 1 (11.1%) | 2 (22.2%) | 0 | 3 (33.3%) |
| D8. Aerosol/whipped cream and cream substitute | 22 | 0 | 10 (45.5%) | 10 (45.5%) | 12 (54.5%) | 0 | 0 | 12 (54.5%) |
| D10. Evaporated/condensed milk | 17 | 0 | 8 (47.1%) | 8 (47.1%) | 2 (11.8%) | 7 (41.2%) | 0 | 9 (52.9%) |
| D11. Milk, buttermilk, milk-based drinks, plant-based milk substitutes^‡^ | 201 | 24 (11.9%) | 114 (56.7%) | 138 (68.7%) | 54 (26.9%) | 9 (4.5%) | 0 | 63 (31.3%) |
| D12. Fermented dairy drinks | 60 | 0 | 8 (13.3%) | 8 (13.3%) | 49 (81.7%) | 3 (5.0%) | 0 | 52 (86.7%) |
| D13. Shakes | 25 | 0 | 7 (28.0%) | 7 (28.0%) | 11 (44.0%) | 6 (24.0%) | 1 (4.0%) | 18 (72.0%) |
| D14. Sour cream | 24 | 0 | 11 (45.8%) | 11 (45.8%) | 13 (54.2%) | 0 | 0 | 13 (54.2%) |
| D15. Yogurt | 340 | 0 | 77 (22.6%) | 77 (22.6%) | 186 (54.7%) | 77 (22.6%) | 0 | 263 (77.4%) |
| **Category D Total** | **1,498** | **24 (1.6%)** | **283 (18.9%)** | **307 (20.5%)** | **666 (44.5%)** | **524 (35.0%)** | **1 (0.1%)** | **1.191 (79.5%)** |
| **E. Desserts** | | | | | | | | |
| E1. Ice cream, frozen yogurt, sherbet, etc. in tubs | 277 | 0 | 0 | 0 | 57 (20.6%) | 218 (78.7%) | 2 (0.7%) | 277 (100.0%) |
| E2 Ice cream, frozen yogurt, sherbet, etc. as cakes, cones | 61 | 0 | 4 (6.6%) | 4 (6.6%) | 14 (23.0%) | 43 (70.5%) | 0 | 57 (93.4%) |
| E3 Ice cream, frozen yogurt, sherbet, etc. as pops, bars | 153 | 0 | 46 (30.1%) | 46 (30.1%) | 49 (32.0%) | 58 (37.9%) | 0 | 107 (69.9%) |
| E4. Sundaes | 8 | 0 | 0 | 0 | 0 | 8 (100.0%) | 0 | 8 (100.0%) |
| E5. Custard, gelatin, pudding | 180 | 0 | 36 (20.0%) | 36 (20.0%) | 124 (68.9%) | 20 (11.1%) | 0 | 144 (80.0%) |
| **Category E Total** | **679** | **0** | **86 (12.7%)** | **86 (12.7%)** | **244 (35.9%)** | **347 (51.1%)** | **2 (0.3%)** | **593 (87.3%)** |
| **F. Dessert Toppings & Fillings** | | | | | | | | |
| F1. Dessert toppings | 29 | 0 | 2 (6.9%) | 2 (6.9%) | 24 (82.8%) | 3 (10.3%) | 0 | 27 (93.1%) |
| F2. Cake frostings | 35 | 0 | 0 | 0 | 20 (57.1%) | 15 (42.9%) | 0 | 35 (100.0%) |
| F3. Pie fillings | 30 | 0 | 5 (16.7%) | 5 (16.7%) | 25 (83.3%) | 0 | 0 | 25 (83.3%) |
| **Category F Total** | **94** | **0** | **7 (7.4%)** | **7 (7.4%)** | **69 (73.4%)** | **18 (19.1%)** | **0** | **87 (92.6%)** |
| **G. Eggs & Substitutes** | | | | | | | | |
| G1. Egg mixtures | 5 | 1 (20.0%) | 1 (20.0%) | 2 (40.0%) | 2 (40.0%) | 0 | 0 | 2 (40.0%) |
| G2. Eggs | 56 | 46 (82.1%) | 8 (14.3%) | 54 (96.4%) | 0 | 2 (3.3%) | 0 | 2 (3.6%) |
| **Category G Total** | **61** | **47 (77.0%)** | **9 (14.8%)** | **56 (91.8%)** | **2 (3.3%)** | **2 (3.3%)** | **0** | **4 (6.2%)** |
| **H. Fats & Oils** | | | | | | | | |
| H1. Butter, margarine, lard, etc.^†^ | 113 | 0 | 34 (30.1%) | 34 (30.1%) | 78 (69.0%) | 1 (0.9%) | 0 | 79 (69.9%) |
| H2. Vegetable oil | 166 | 0 | 153 (92.2%) | 153 (92.2%) | 13 (7.8%) | 0 | 0 | 13 (7.8%) |
| H4. Dressings for salad | 285 | 0 | 83 (29.1%) | 83 (29.1%) | 188 (66.0%) | 14 (4.9%) | 0 | 202 (70.9%) |
| H5. Mayonnaise & mayonnaise-type dressings | 69 | 0 | 47 (68.1%) | 47 (68.1%) | 22 (31.9%) | 0 | 0 | 22 (31.9%) |
| H6. Spray oil | 23 | 0 | 23 (100.0%) | 23 (100.0%) | 0 | 0 | 0 | 0 |
| **Category H Total** | **656** | **0** | **340 (51.8%)** | **340 (51.8%)** | **301 (45.9%)** | **15 (2.3%)** | **0** | **316 (48.2%)** |
| **I. Fish, Seafood & Substitutes** | | | | | | | | |
| I1. Anchovies, caviar | 9 | 0 | 3 (33.3%) | 3 (33.3%) | 6 (66.7%) | 0 | 0 | 6 (66.7%) |
| I2. Marine & freshwater animals with sauce^†^ | 50 | 0 | 7 (14.0%) | 7 (14.0%) | 29 (58.0%) | 11 (22.0%) | 3 (6.0%) | 43 (86.0%) |
| I3. Marine & freshwater animals without sauce | 191 | 0 | 73 (38.2%) | 73 (38.2%) | 95 (49.7%) | 23 (12.0%) | 0 | 118 (61.8%) |
| I4. Canned marine and freshwater animals | 147 | 0 | 117 (79.6%) | 117 (79.6%) | 26 (17.7%) | 4 (2.7%) | 0 | 30 (20.4%) |
| I5. Smoked/pickled marine & freshwater animals | 49 | 0 | 13 (26.5%) | 13 (26.5%) | 34 (69.4%) | 2 (4.1%) | 0 | 36 (73.5%) |
| **Category I Total** | **446** | **0** | **213 (47.8%)** | **213 (47.8%)** | **190 (42.6%)** | **40 (9.0%)** | **3 (0.7%)** | **233 (52.2%)** |
| **J. Fruits & Fruit Juices** | | | | | | | | |
| J1. Fruits (fresh, frozen, canned, coated, and uncoated) ^‡^ | 194 | 66 (34.0%) | 37 (19.1%) | 103 (53.1%) | 91 (46.9%) | 0 | 0 | 91 (46.9%) |
| J2. Berries | 15 | 15 (100.0%) | 0 | 15 (100.0%) | 0 | 0 | 0 | 0 |
| J3. Melons | 7 | 7 (100.0%) | 0 | 7 (100.0%) | 0 | 0 | 0 | 0 |
| J4. Avocados | 1 | 0 | 1 (100.0%) | 1 (100.0%) | 0 | 0 | 0 | 0 |
| J5. Fruits used as ingredients | 3 | 3 (100.0%) | 0 | 3 (100.0%) | 0 | 0 | 0 | 0 |
| J6. Apple sauces | 65 | 11 (16.9%) | 35 (53.8%) | 46 (70.8%) | 19 (29.2%) | 0 | 0 | 19 (29.2%) |
| J7. Dried fruits^‡^ | 132 | 57 (43.2%) | 7 (5.3%) | 64 (48.5%) | 65 (49.2%) | 3 (2.3%) | 0 | 68 (51.5%) |
| J8. Candied/pickled fruits | 21 | 0 | 0 | 0 | 21 (100.0%) | 0 | 0 | 21 (100.0%) |
| J9. Fruits for garnish | 5 | 0 | 5 (100.0%) | 5 (100.0%) | 0 | 0 | 0 | 0 |
| J11. Juices, nectars, fruit drinks^‡^ | 608 | 0 | 60 (9.9%) | 60 (9.9%) | 544 (89.5%) | 4 (0.7%) | 0 | 548 (90.1%) |
| J12. Fruit juices used as ingredients^§^ | 10 | 0 | 10 (100.0%) | 10 (100.0%) | 0 | 0 | 0 | 0 |
| **Category J Total** | **1,061** | **159 (15.0%)** | **155 (14.6%)** | **314 (29.6%)** | **740 (69.7%)** | **7 (0.7%)** | **0** | **747 (70.4%)** |
| **K. Legumes** | | | | | | | | |
| K1. Tofu or tempeh | 23 | 0 | 17 (73.9%) | 17 (73.9%) | 6 (26.1%) | 0 | 0 | 6 (26.1%) |
| K2. Beans, lentils, peas, etc. | 165 | 0 | 149 (90.3%) | 149 (90.3%) | 16 (9.7%) | 0 | 0 | 16 (9.7%) |
| **Category K Total** | **188** | **0** | **166 (88.3%)** | **166 (88.3%)** | **22 (11.7%)** | **0** | **0** | **22 (11.7%)** |
| **L. Meats & Substitutes** | | | | | | | | |
| L1. Pork rinds and bacon | 41 | 0 | 0 | 0 | 18 (43.9%) | 23 (56.1%) | 0 | 41 (100.0%) |
| L2. Beef, pork and poultry breakfast strips | 6 | 0 | 1 (16.7%) | 1 (16.7%) | 4 (66.7%) | 1 (16.7%) | 0 | 5 (83.3%) |
| L3. Dried meat & poultry^†^ | 97 | 0 | 0 | 0 | 21 (21.6%) | 76 (78.4%) | 0 | 97 (100.0%) |
| L4. Luncheon meats | 85 | 0 | 4 (4.7%) | 4 (4.7%) | 56 (65.9%) | 25 (29.4%) | 0 | 81 (95.3%) |
| L5. Sausage products | 160 | 0 | 4 (2.5%) | 4 (2.5%) | 38 (23.8%) | 118 (73.8%) | 0 | 156 (97.5%) |
| L6. Cust of meat & poultry without sauce^‡^ | 130 | 0 | 17 (13.1%) | 17 (13.1%) | 77 (59.2%) | 36 (27.7%) | 0 | 113 (86.9%) |
| L7. Patties, ground meats with and without breading | 214 | 0 | 38 (17.8%) | 38 (17.8%) | 105 (49.1%) | 71 (33.2%) | 0 | 176 (82.2%) |
| L8. Cured meats^†^ | 86 | 0 | 3 (3.5%) | 3 (3.5%) | 68 (79.1%) | 15 (17.4%) | 0 | 83 (96.5%) |
| L9. Canned meats | 27 | 0 | 3 (11.1%) | 3 (11.1%) | 10 (37.0%) | 14 (51.9%) | 0 | 24 (88.9%) |
| L10. Meat and poultry with sauce | 116 | 0 | 1 (0.9%) | 1 (0.9%) | 33 (28.4%) | 66 (56.9%) | 16 (13.8%) | 115 (99.1%) |
| **Category L Total** | **962** | **0** | **71 (7.4%)** | **71 (7.4%)** | **430 (44.7%)** | **445 (46.3%)** | **16 (1.7%)** | **891 (92.6%)** |
| **M. Miscellaneous** | | | | | | | | |
| M1. Baking powder, baking soda, yeast^‡^ | 25 | 0 | 19 (76.0%) | 19 (76.0%) | 6 (24.0%) | 0 | 0 | 6 (24.0%) |
| M2. Baking decorations^§^ | 20 | 0 | 20 (100.0%) | 20 (100.0%) | 0 | 0 | 0 | 0 |
| M3. Breadcrumbs | 244 | 0 | 72 (29.5%) | 72 (29.5%) | 132 (54.1%) | 39 (16.0%) | 1 (0.4%) | 172 (70.5%) |
| M4. Cooking wine | 1 | 0 | 1 (100.0%) | 1 (100.0%) | 0 | 0 | 0 | 0 |
| M5. Cocoa powder | 5 | 0 | 5 (100.0%) | 5 (100.0%) | 0 | 0 | 0 | 0 |
| M7. Chewing gum | 3 | 0 | 3 (100.0%) | 3 (100.0%) | 0 | 0 | 0 | 0 |
| M8. Salad & potato toppers | 23 | 0 | 11 (47.8%) | 11 (47.8%) | 11 (47.8%) | 1 (4.3%) | 0 | 12 (52.2%) |
| M9. Salt, salt substitutes^†,‡^ | 167 | 4 (2.4%) | 34 (20.4%) | 38 (22.8%) | 127 (76.0%) | 1 (0.6%) | 1 (0.6%) | 129 (77.2%) |
| M10. Spices and herbs without salt | 36 | 0 | 36 (100.0%) | 36 (100.0%) | 0 | 0 | 0 | 0 |
| M11. Coconut milk | 19 | 0 | 0 | 0 | 19 (100.0%) | 0 | 0 | 19 (100.0%) |
| M12. Dried coconut | 14 | 0 | 0 | 0 | 6 (42.9%) | 8 (57.1%) | 0 | 14 (100.0%) |
| **Category M Total** | **557** | **4 (0.7%)** | **201 (36.1%)** | **205 (36.8%)** | **301 (54.0%)** | **49 (8.8%)** | **2 (0.4%)** | **352 (63.2%)** |
| **N. Combination Dishes** | | | | | | | | |
| N1. Combination dishes | 547 | 0 | 3 (0.5%) | 3 (0.5%) | 197 (36.0%) | 330 (60.3%) | 17 (3.1%) | 544 (99.5%) |
| N2. Burritos, pizzas, sandwiches, etc.^‡^ | 467 | 0 | 5 (1.1%) | 5 (1.1%) | 110 (23.6%) | 330 (70.7%) | 22 (4.7%) | 462 (98.9%) |
| N3. Hors d’oeuvres | 126 | 0 | 13 (10.3%) | 13 (10.3%) | 72 (57.1%) | 39 (31.0%) | 2 (1.6%) | 113 (89.7%) |
| **Category N Total** | **1,140** | **0** | **21 (1.8%)** | **21 (1.8%)** | **379 (33.2%)** | **699 (61.3%)** | **41 (3.6%)** | **1119 (98.2%)** |
| **O. Nuts & Seeds** | | | | | | | | |
| O1. Nuts & seeds (not used for snacks) ^‡^ | 143 | 0 | 85 (59.4%) | 85 (59.4%) | 58 (40.6%) | 0 | 0 | 58 (40.6%) |
| O2. Nut pastes and creams | 7 | 0 | 0 | 0 | 5 (71.4%) | 2 (28.6%) | 0 | 7 (100.0%) |
| O3. Nut butters | 101 | 0 | 82 (81.2%) | 82 (81.2%) | 19 (18.8%) | 0 | 0 | 19 (18.8%) |
| O4. Nut flours | 4 | 0 | 2 (50.0%) | 2 (50.0%) | 2 (50.0%) | 0 | 0 | 2 (50.0%) |
| **Category O Total** | **255** | **0** | **169 (66.3%)** | **169 (66.3%)** | **84 (32.9%)** | **2 (0.8%)** | **0** | **86 (33.7%)** |
| **P. Potatoes** | | | | | | | | |
| P1. French fries | 65 | 0 | 52 (80.0%) | 52 (80.0%) | 13 (20.0%) | 0 | 0 | 13 (20.0%) |
| P2. Mashed, stuffed, candied potatoes | 37 | 0 | 5 (13.5%) | 5 (13.5%) | 18 (48.6%) | 14 (37.8%) | 0 | 32 (86.5%) |
| P3. Fresh, canned, frozen potatoes | 30 | 0 | 22 (73.3%) | 22 (73.3%) | 8 (26.7%) | 0 | 0 | 8 (26.7%) |
| **Category P Total** | **132** | **0** | **79 (59.8%)** | **79 (59.8%)** | **39 (29.5%)** | **14 (10.6%)** | **0** | **53 (40.2%)** |
| **Q. Salads** | | | | | | | | |
| Q1. Salads (including egg, fish, beans, vegetables) ^†,‡^ | 109 | 0 | 51 (46.8%) | 51 (46.8%) | 41 (37.6%) | 17 (15.6%) | 0 | 58 (53.2%) |
| Q3. Pasta/potato salad | 21 | 0 | 6 (28.6%) | 6 (28.6%) | 8 (38.1%) | 7 (33.3%) | 0 | 15 (71.4%) |
| **Category Q Total** | **130** | **0** | **57 (43.8%)** | **57 (43.8%)** | **49 (37.7%)** | **24 (18.5%)** | **0** | **73 (56.2%)** |
| **R. Sauces & Dips** | | | | | | | | |
| R1. Dipping sauces | 158 | 0 | 25 (15.8%) | 25 (15.8%) | 56 (35.4%) | 77 (48.7%) | 0 | 133 (84.2%) |
| R2. Dips and spreads | 186 | 0 | 142 (76.3%) | 142 (76.3%) | 38 (20.4%) | 6 (3.2%) | 0 | 44 (23.7%) |
| R3. Major main entrée sauce | 226 | 0 | 41 (18.1%) | 41 (18.1%) | 138 (61.1%) | 46 (20.4%) | 1 (0.4%) | 185 (81.9%) |
| R4. Minor main entrée sauce | 264 | 0 | 127 (48.1%) | 127 (48.1%) | 107 (40.5%) | 30 (11.4%) | 0 | 137 (51.9%) |
| R5. Major condiments^‡,§^ | 295 | 0 | 72 (24.4%) | 72 (24.4%) | 195 (66.1%) | 28 (9.5%) | 0 | 223 (75.6%) |
| R6. Minor condiments^†,‡^ | 121 | 0 | 85 (70.2%) | 85 (70.2%) | 36 (29.8%) | 0 | 0 | 36 (29.8%) |
| **Category R Total** | **1,250** | **0** | **492 (39.4%)** | **492 (39.4%)** | **570 (45.6%)** | **187 (15.0%)** | **1 (0.1%)** | **758 (60.6%)** |
| **S. Snacks** | | | | | | | | |
| S1. Chips, pretzels, etc. | 577 | 0 | 287 (49.7%) | 287 (49.7%) | 235 (40.7%) | 50 (8.7%) | 5 (0.9%) | 290 (50.3%) |
| S2. Nuts or seeds (used as snacks) | 258 | 0 | 53 (20.5%) | 53 (20.5%) | 176 (68.2%) | 29 (11.2%) | 0 | 205 (79.5%) |
| S3. Meat/poultry snack food sticks | 31 | 0 | 0 | 0 | 7 (22.6%) | 24 (77.4%) | 0 | 31 (100.0%) |
| **Category S Total** | **866** | **0** | **340 (39.3%)** | **340 (39.3%)** | **418 (48.3%)** | **103 (11.9%)** | **5 (0.6%)** | **526 (60.7%)** |
| **T. Soups** | | | | | | | | |
| T1. All varieties of soups (includes broth) | 480 | 0 | 28 (5.8%) | 28 (5.8%) | 340 (70.8%) | 109 (22.7%) | 3 (0.6%) | 452 (94.2%) |
| **Category T Total** | 480 | **0** | **28 (5.8%)** | **28 (5.8%)** | **340 (70.8%)** | **109 (22.7%)** | **3 (0.6%)** | **452 (94.2%)** |
| **U. Sugars & Sweets** | | | | | | | | |
| U1. Candies, confectionaries, chocolates^‡^ | 541 | 0 | 5 (0.9%) | 5 (0.9%) | 220 (40.7%) | 316 (58.4%) | 0 | 536 (99.1%) |
| U3. Hard candies | 20 | 0 | 11 (55.0%) | 11 (55.0%) | 9 (45.0%) | 0 | 0 | 9 (45.0%) |
| U4. Baking candies | 47 | 0 | 0 | 0 | 6 (12.8%) | 41 (87.2%) | 0 | 47 (100.0%) |
| U5. Breath mints | 2 | 0 | 2 (100.0%) | 2 (100.0%) | 0 | 0 | 0 | 0 |
| U7. Icing sugar | 3 | 3 (100.0%) | 0 | 3 (100.0%) | 0 | 0 | 0 | 0 |
| U8. Honey, molasses, bread spreads | 72 | 58 (80.6%) | 0 | 58 (80.6%) | 9 (12.5%) | 5 (6.9%) | 0 | 14 (19.4%) |
| U9. Jams, jellies, fruit spreads^‡^ | 228 | 0 | 23 (10.1%) | 23 (10.1%) | 203 (89.0%) | 2 (0.9%) | 0 | 205 (89.9%) |
| U10. Fruit leather | 20 | 0 | 0 | 0 | 19 (95.0%) | 1 (5.0%) | 0 | 20 (100.0%) |
| U11. Marshmallows | 12 | 0 | 0 | 0 | 12 (100.0%) | 0 | 0 | 12 (100.0%) |
| U12. Sugars^†,‡,§^ | 45 | 45 (100.0%) | 0 | 45 (100.0%) | 0 | 0 | 0 | 0 |
| U14. Syrups used as toppings | 52 | 52 (100.0%) | 0 | 52 (100.0%) | 0 | 0 | 0 | 0 |
| U15. Syrups used as ingredients | 20 | 20 (100.0%) | 0 | 20 (100.0%) | 0 | 0 | 0 | 0 |
| **Category U Total** | **1,062** | **178 (16.8%)** | **41 (3.9%)** | **219 (20.6%)** | **478 (45.0%)** | **365 (34.4%)** | **0** | **843 (79.4%)** |
| **V. Vegetables** | | | | | | | | |
| V1. Vegetables without sauce^‡^ | 411 | 226 (55.0%) | 175 (42.6%) | 401 (97.6%) | 10 (2.4%) | 0 | 0 | 10 (2.4%) |
| V2. Vegetables with sauce | 13 | 0 | 6 (46.2%) | 6 (46.2%) | 3 (23.1%) | 4 (30.8%) | 0 | 7 (53.8%) |
| V3. Vegetables used for garnishing/flavouring^†,‡^ | 27 | 3 (11.1%) | 20 (74.1%) | 23 (85.2%) | 4 (14.8%) | 0 | 0 | 4 (14.8%) |
| V4. Chili pepper & green onion | 42 | 3 (7.1%) | 15 (35.7%) | 18 (42.9%) | 24 (57.1%) | 0 | 0 | 24 (57.1%) |
| V5. Seaweed, dehydrated mushrooms | 19 | 12 (63.2%) | 1 (5.3%) | 13 (68.4%) | 6 (31.6%) | 0 | 0 | 6 (31.6%) |
| V6. Sprouts | 1 | 1 (100.0%) | 0 | 1 (100.0%) | 0 | 0 | 0 | 0 |
| V7. Vegetable juice and drink | 60 | 0 | 17 (28.3%) | 17 (28.3%) | 43 (71.7%) | 0 | 0 | 43 (71.7%) |
| V8. Olives | 65 | 0 | 4 (6.2%) | 4 (6.2%) | 59 (90.8%) | 2 (3.1%) | 0 | 61 (93.8%) |
| V9. Sun-dried tomatoes and other pickled or oil-packed vegetables^‡,§^ | 172 | 1 (0.6%) | 66 (38.4%) | 67 (39.0%) | 100 (58.1%) | 5 (2.9%) | 0 | 105 (61.0%) |
| V10. Relish | 15 | 0 | 11 (73.3%) | 11 (73.3%) | 3 (20.0%) | 1 (6.7%) | 0 | 4 (26.7%) |
| V11. Vegetable paste | 12 | 5 (41.7%) | 7 (58.3%) | 12 (100.0%) | 0 | 0 | 0 | 0 |
| V12. Vegetable sauce or purée^‡^ | 34 | 3 (8.8%) | 26 (76.5%) | 29 (85.3%) | 5 (14.7%) | 0 | 0 | 5 (14.7%) |
| **Category V Total** | **871** | **254 (29.2%)** | **348 (40.0%)** | **602 (69.1%)** | **257 (29.5%)** | **12 (1.4%)** | **0** | **269 (30.9%)** |
| **W. Foods for <4 years old** | | | | | | | | |
| W1. Cereals to be prepared^‡^ | 41 | 0 | 21 (51.2%) | 21 (51.2%) | 20 (48.8%) | 0 | 0 | 20 (48.8%) |
| W2. Ready-to-eat cereals and cereal bars^‡^ | 9 | 0 | 0 | 0 | 9 (100.0%) | 0 | 0 | 9 (100.0%) |
| W3. Cookies, biscuits, etc.^‡^ | 47 | 0 | 41 (87.2%) | 41 (87.2%) | 6 (12.8%) | 0 | 0 | 6 (12.8%) |
| W4. Strained meat, desserts, combination dishes^‡^ | 124 | 0 | 61 (49.2%) | 61 (49.2%) | 63 (50.8%) | 0 | 0 | 63 (50.8%) |
| W5. Combination dishes^‡^ | 9 | 0 | 6 (66.7%) | 6 (66.7%) | 3 (33.3%) | 0 | 0 | 3 (33.3%) |
| W6. Juices^‡^ | 2 | 0 | 0 | 0 | 2 (100.0%) | 0 | 0 | 2 (100.0%) |
| **Category W Total** | **232** | **0** | **129 (55.6%)** | **129 (55.6%)** | **103 (44.4%)** | **0** | **0** | **103 (44.4%)** |
| **OVERALL TOTAL** | **17,521** | **666 (3.8%)** | **5,662 (32.3%)** | **6,328 (36.1%)** | **7,296 (41.6%)** | **3,733 (21.3%)** | **164 (0.9%)** | **11,193 (63.9%)** |

All values are presented as n (%); n=17,521. ^*^Major and minor categories were defined as per Health Canada’s Table of Reference Amounts for Foods [2]. ^†^Indicates categories with products that were missing values for total sugars (n=13; 0.1%, overall). ^‡^Indicates categories with products that were missing values for saturated fats (n=299; 1.7%, overall). ^§^Indicates categories with products that were missing values for sodium (n=11; 0.1%, overall). Abbreviations: FLIP, Food Label Information Program; FOP, front-of-pack; TRA, Table of Reference Amounts for Foods.

# **Supplementary Table 7.** Summary of the nutrient types that would be displayed in a FOP symbol in FLIP2017 based on the proposed FOP labelling regulations

|  |  | **FOP Symbol “High in” Nutrient Type** | | |
| --- | --- | --- | --- | --- |
| **TRA Category^*^** | **n** | **Total Sugars** | **Saturated Fat** | **Sodium** |
| **A. Bakery Products** | | | | |
| A1. Bread, excluding sweet quick-type | 340 | 3 (0.9%) | 5 (1.5%) | 151 (44.4%) |
| A2. Tea biscuits, scones, rolls, buns, etc. | 290 | 2 (0.7%) | 42 (14.5%) | 92 (31.7%) |
| A3. Bagels, naan, flat bread | 86 | 0 | 3 (3.5%) | 61 (70.9%) |
| A4. Brownies | 29 | 24 (82.8%) | 15 (51.7%) | 0 |
| A5. Heavy weight cake | 68 | 68 (100.0%) | 67 (98.5%) | 21 (30.9%) |
| A6. Medium weight cake | 155 | 145 (93.5%) | 142 (91.6%) | 33 (21.3%) |
| A7. Light weight cake | 5 | 5 (100.0%) | 0 | 1 (20.0%) |
| A8. Coffee cakes, doughnuts, sweet quick-type breads, etc. | 111 | 64 (57.7%) | 61 (55.0%) | 14 (12.6%) |
| A9. Muffins | 40 | 39 (97.5%) | 33 (82.5%) | 13 (32.5%) |
| A10. Cookies | 523 | 335 (64.1%) | 351 (67.1%)^†^ | 1 (0.2%) |
| A11. Accompaniment crackers | 254 | 0 | 36 (14.2%)^†^ | 90 (35.4%) |
| A12. Snack crackers | 66 | 2 (3.0%) | 8 (12.1%) | 48 (72.7%) |
| A13. Dry breads | 88 | 6 (6.8%) | 8 (9.1%) | 33 (37.5%) |
| A14. Toaster pastries | 11 | 5 (45.5%) | 1 (9.1%) | 0 |
| A15. Ice cream cones | 20 | 1 (5.0%) | 1 (5.0%) | 0 |
| A16. Croutons | 37 | 0 | 1 (2.7%) | 3 (8.1%) |
| A17. French toast, pancakes, waffles | 54 | 2 (3.7%) | 2 (3.7%) | 45 (83.3%) |
| A18. Grain-based bars with filling | 105 | 56 (53.3%) | 42 (40.0%) | 0 |
| A19. Grain-based bars without filling | 94 | 26 (27.7%) | 10 (10.6%) | 1 (1.1%) |
| A20. Energy and protein bars | 205 | 65 (31.7%) | 96 (46.8%) | 1 (0.5%) |
| A21. Rice and corn cakes | 35 | 2 (5.7%) | 2 (5.7%) | 18 (51.4%) |
| A22. Pies, tarts, etc. | 114 | 101 (88.6%) | 107 (93.9%) | 20 (17.5%) |
| A23. Pie crust | 18 | 1 (5.6%) | 18 (100.0%) | 0 |
| A24. Pizza crust | 14 | 0 | 0 | 3 (21.4%) |
| A25. Taco shell | 13 | 0 | 6 (46.2%) | 2 (15.4%) |
| **Category A Total** | **2,775** | **952 (34.3%)** | **1,057 (38.1%)** | **651 (23.5%)** |
| **B. Beverages** | | | | |
| B1. Carbonated and non-carbonated beverages | 718 | 369 (51.4%)^†^ | 13 (1.8%)^†^ | 13 (1.8%) |
| B3. Coffee | 28 | 3 (10.7%)^†^ | 5 (17.9%)^†^ | 0^†^ |
| B4. Tea | 73 | 7 (9.6%) | 3 (4.1%) | 0 |
| B5. Cocoa and hot chocolate beverages | 32 | 24 (75.0%) | 12 (37.5%) | 0 |
| **Category B Total** | **851** | **403 (47.4%)** | **33 (3.9%)** | **13 (1.5%)** |
| **C. Cereals & Other Grains** | | | | |
| C1. Hot breakfast cereals | 118 | 15 (12.7%) | 1 (0.8%) | 0 |
| C2. Ready-to-eat cereals, puffed and coated | 4 | 0 | 0 | 0 |
| C3. Ready-to-eat cereals, fruit and nut type | 88 | 32 (36.4%) | 0 | 9 (10.2%) |
| C4. Ready-to-eat cereals, granola type | 169 | 24 (14.2%) | 19 (11.2%)^†^ | 0 |
| C5. Bran and wheat germ, milled flax, etc. | 38 | 0 | 1 (2.6%) | 0 |
| C6. Flours and cornmeal | 66 | 0 | 0 | 2 (3.0%) |
| C7. Grains | 278 | 0 | 2 (0.7%) | 80 (28.8%) |
| C8. Pastas | 493 | 1 (0.2%) | 49 (9.9%) | 66 (13.4%) |
| C10. Starch | 11 | 0 | 0 | 2 (18.2%) |
| C11. Stuffing | 10 | 0 | 4 (40.0%) | 8 (80.0%) |
| **Category C Total** | **1,275** | **72 (5.6%)** | **76 (6.0%)** | **167 (13.1%)** |
| **D. Dairy Products & Substitutes** | | | | |
| D1. Cheese | 569 | 1 (0.2%) | 553 (97.2%)^†^ | 312 (54.8%) |
| D2. Cottage cheese | 21 | 1 (4.8%) | 1 (4.8%) | 0 |
| D3. Cheese used as ingredients | 14 | 0 | 7 (50.0%) | 0 |
| D4. Hard cheese | 45 | 0 | 45 (100.0%) | 33 (73.3%) |
| D5. Quark | 110 | 5 (4.5%) | 106 (96.4%) | 77 (70.0%) |
| D6. Cream and cream substitutes | 41 | 15 (36.6%) | 22 (53.7%) | 0 |
| D7. Powder cream and cream substitute | 9 | 2 (22.2%) | 3 (33.3%) | 0 |
| D8. Aerosol/whipped cream and cream substitutes | 22 | 0 | 12 (54.5%) | 0 |
| D10. Evaporated/condensed milk | 17 | 8 (47.1%) | 8 (47.1%) | 0 |
| D11. Milk, buttermilk, milk-based drinks, plant-based milk substitutes | 201 | 45 (22.4%)^†^ | 27 (13.4%) | 0 |
| D12. Fermented dairy drinks | 60 | 48 (80.0%) | 6 (10.0%) | 1 (1.7%) |
| D13. Shakes | 25 | 18 (72.0%) | 7 (28.0%) | 1 (4.0%) |
| D14. Sour cream | 24 | 0 | 13 (54.2%) | 0 |
| D15. Yogurt | 340 | 242 (71.2%) | 98 (28.8%) | 0 |
| **Category D Total** | **1,498** | **385 (25.7%)** | **908 (60.6%)** | **424 (28.3%)** |
| **E. Desserts** | | | | |
| E1. Ice cream, frozen yogurt, sherbet, etc. in tubs | 277 | 273 (98.6%) | 224 (80.9%) | 2 (0.7%) |
| E2 Ice cream, frozen yogurt, sherbet, etc. as cakes, cones | 61 | 52 (85.2%) | 48 (78.7%) | 0 |
| E3 Ice cream, frozen yogurt, sherbet, etc. as pops, bars | 153 | 84 (54.9%) | 81 (52.9%) | 0 |
| E4. Sundaes | 8 | 8 (100.0%) | 8 (100.0%) | 0 |
| E5. Custard, gelatin, pudding | 180 | 134 (74.4%) | 19 (10.6%) | 11 (6.1%) |
| **Category E Total** | **679** | **551 (81.1%)** | **380 (56.0%)** | **13 (1.9%)** |
| **F. Dessert Toppings & Fillings** | | | | |
| F1. Dessert toppings | 29 | 27 (93.1%) | 3 (10.3%) | 0 |
| F2. Cake frostings | 35 | 35 (100.0%) | 15 (42.9%) | 0 |
| F3. Pie fillings | 30 | 25 (83.3%) | 0 | 0 |
| **Category F Total** | **94** | **87 (92.6%)** | **18 (19.1%)** | **0** |
| **G. Eggs & Substitutes** | | | | |
| G1. Egg mixtures | 5 | 0 | 1 (20.0%) | 3 (60.0%) |
| G2. Eggs | 56 | 0 | 2 (3.6%) | 2 (3.6%) |
| **Category G Total** | **61** | **0** | **3 (4.9%)** | **5 (8.2%)** |
| **H. Fats & Oils** | | | | |
| H1. Butter, margarine, lard, etc. | 113 | 0^†^ | 79 (69.9%) | 1 (0.9%) |
| H2. Vegetable oil^†^ | 166 | 0 | 13 (7.8%) | 0 |
| H4. Dressings for salad | 285 | 7 (2.5%) | 13 (4.6%) | 196 (68.8%) |
| H5. Mayonnaise & mayonnaise-type dressings | 69 | 0 | 0 | 22 (31.9%) |
| H6. Spray oil | 23 | 0 | 0 | 0 |
| **Category H Total** | **656** | **7 (1.1%)** | **105 (16.0%)** | **219 (33.4%)** |
| **I. Fish, Seafood & Substitutes** | | | | |
| I1. Anchovies, caviar | 9 | 0 | 0 | 6 (66.7%) |
| I2. Marine & freshwater animals with sauce^†^ | 50 | 3 (6.0%) | 15 (30.0%) | 42 (84.0%) |
| I3. Marine & freshwater animals without sauce | 191 | 0 | 29 (15.2%) | 112 (58.6%) |
| I4. Canned marine and freshwater animals | 147 | 0 | 7 (4.8%) | 27 (18.4%) |
| I5. Smoked/pickled marine & freshwater animals | 49 | 0 | 8 (16.3%) | 30 (61.2%) |
| **Category I Total** | **446** | **3 (0.7%)** | **59 (13.2%)** | **217 (48.7%)** |
| **J. Fruits & Fruit Juices** | | | | |
| J1. Fruits (fresh, frozen, canned, coated, and uncoated) | 194 | 88 (45.4%) | 3 (1.5%) | 0 |
| J2. Berries | 15 | 0 | 0 | 0 |
| J3. Melons | 7 | 0 | 0 | 0 |
| J4. Avocados | 1 | 0 | 0 | 0 |
| J5. Fruits used as ingredients | 3 | 0 | 0 | 0 |
| J6. Apple sauces | 65 | 19 (29.2%) | 0 | 0 |
| J7. Dried fruits | 132 | 68 (51.5%) | 3 (2.3%)^†^ | 0 |
| J8. Candied/pickled fruits | 21 | 12 (57.1%) | 0 | 9 (42.9%) |
| J9. Fruits for garnish | 5 | 0 | 0 | 0 |
| J11. Juices, nectars, fruit drinks | 608 | 547 (90.0%) | 3 (0.5%)^†^ | 2 (0.3%) |
| J12. Fruit juices used as ingredients | 10 | 0 | 0 | 0^†^ |
| **Category J Total** | **1,061** | **734 (69.2%)** | **9 (0.8%)** | **11 (1.0%)** |
| **K. Legumes** | | | | |
| K1. Tofu or tempeh | 23 | 5 (21.7%) | 0 | 1 (4.3%) |
| K2. Beans, lentils, peas, etc. | 165 | 0 | 0 | 16 (9.7%) |
| **Category K Total** | **188** | **5 (2.7%)** | **0** | **17 (9.0%)** |
| **L. Meats & Substitutes** | | | | |
| L1. Pork rinds and bacon | 41 | 0 | 40 (97.6%) | 24 (58.5%) |
| L2. Beef, pork and poultry breakfast strips | 6 | 0 | 1 (16.7%) | 5 (83.3%) |
| L3. Dried meat and poultry | 97 | 8 (8.2%)^†^ | 68 (70.1%) | 97 (100.0%) |
| L4. Luncheon meats | 85 | 0 | 30 (35.3%) | 76 (89.4%) |
| L5. Sausage products | 160 | 0 | 119 (74.4%) | 155 (96.9%) |
| L6. Cust of meat & poultry without sauce | 130 | 2 (1.5%) | 54 (41.5%)^†^ | 93 (71.5%) |
| L7. Patties, ground meats with and without breading | 214 | 0 | 111 (51.9%) | 136 (63.6%) |
| L8. Cured meats | 86 | 0^†^ | 15 (17.4%) | 83 (96.5%) |
| L9. Canned meats | 27 | 0 | 16 (59.3%) | 22 (81.5%) |
| L10. Meat and poultry with sauce | 116 | 37 (31.9%) | 63 (54.3%) | 113 (97.4%) |
| **Category L Total** | **962** | **47 (4.9%)** | **517 (53.7%)** | **804 (83.6%)** |
| **M. Miscellaneous** | | | | |
| M1. Baking powder, baking soda, yeast | 25 | 2 (8.0%) | 0 | 4 (16.0%)^†^ |
| M2. Baking decorations | 20 | 0^†^ | 0 | 0 |
| M3. Breadcrumbs | 244 | 105 (43.0%) | 40 (16.4%) | 68 (27.9%) |
| M4. Cooking wine | 1 | 0 | 0 | 0 |
| M5. Cocoa powder | 5 | 0 | 0 | 0 |
| M7. Chewing gum | 3 | 0 | 0 | 0 |
| M8. Salad & potato toppers | 23 | 0 | 8 (34.8%) | 5 (21.7%) |
| M9. Salt, salt substitutes | 167 | 1 (0.6%) | 2 (1.2%)^†^ | 129 (77.2%)^†^ |
| M10. Spices and herbs without salt | 36 | 0 | 0 | 0 |
| M11. Coconut milk | 19 | 0 | 19 (100.0%) | 0 |
| M12. Dried coconut | 14 | 8 (57.1%) | 14 (100.0%) | 0 |
| **Category M Total** | **557** | **116 (20.8%)** | **83 (14.9%)** | **206 (37.0%)** |
| **N. Combination Dishes** | | | | |
| N1. Combination dishes | 547 | 84 (15.4%) | 286 (52.3%) | 538 (98.4%) |
| N2. Burritos, pizzas, sandwiches, etc. | 467 | 30 (6.4%) | 346 (74.1%)^†^ | 460 (98.5%) |
| N3. Hors d’oeuvres | 126 | 5 (4.0%) | 54 (42.9%) | 97 (77.0%) |
| **Category N Total** | **1,140** | **119 (10.4%)** | **686 (60.2%)** | **1,095 (96.1%)** |
| **O. Nuts & Seeds** | | | | |
| O1. Nuts & seeds (not used for snacks) | 143 | 0 | 58 (40.6%)^†^ | 0 |
| O2. Nut pastes and creams | 7 | 7 (100.0%) | 2 (28.6%) | 0 |
| O3. Nut butters | 101 | 1 (1.0%) | 18 (17.8%) | 0 |
| O4. Nut flours | 4 | 0 | 2 (50.0%) | 0 |
| **Category O Total** | **255** | **8 (3.1%)** | **80 (31.4%)** | **0** |
| **P. Potatoes** | | | | |
| P1. French fries | 65 | 0 | 1 (1.5%) | 12 (18.5%) |
| P2. Mashed, stuffed, candied potatoes | 37 | 0 | 15 (40.5%) | 31 (83.8%) |
| P3. Fresh, canned, frozen potatoes | 30 | 4 (13.3%) | 0 | 4 (13.3%) |
| **Category P Total** | **132** | **4 (3.0%)** | **16 (12.1%)** | **47 (35.6%)** |
| **Q. Salads** | | | | |
| Q1. Salads (including egg, fish, beans, vegetables) | 109 | 10 (9.2%)^†^ | 21 (19.3%)^†^ | 44 (40.4%) |
| Q3. Pasta/potato salad | 21 | 0 | 7 (33.3%) | 15 (71.4%) |
| **Category Q Total** | **130** | **10 (7.7%)** | **28 (21.5%)** | **59 (45.4%)** |
| **R. Sauces & Dips** | | | | |
| R1. Dipping sauces | 158 | 98 (62.0%) | 4 (2.5%) | 108 (68.4%) |
| R2. Dips and spreads | 186 | 1 (0.5%) | 26 (14.0%) | 23 (12.4%) |
| R3. Major main entrée sauce | 226 | 10 (4.4%) | 45 (19.9%) | 178 (78.8%) |
| R4. Minor main entrée sauce | 264 | 28 (10.6%) | 35 (13.3%) | 104 (39.4%) |
| R5. Major condiments | 295 | 72 (24.4%) | 0^†^ | 179 (60.7%)^†^ |
| R6. Minor condiments | 121 | 0^†^ | 0^†^ | 36 (29.8%) |
| **Category R Total** | **1,250** | **209 (16.7%)** | **110 (8.8%)** | **628 (50.2%)** |
| **S. Snacks** | | | | |
| S1. Chips, pretzels, etc. | 577 | 33 (5.7%) | 115 (19.9%)^†^ | 202 (35.0%) |
| S2. Nuts or seeds (used as snacks) | 258 | 53 (20.5%) | 168 (65.1%)^†^ | 13 (5.0%) |
| S3. Meat/poultry snack food sticks | 31 | 0 | 24 (77.4%) | 31 (100.0%) |
| **Category S Total** | **866** | **86 (9.9%)** | **307 (35.5%)** | **246 (28.4%)** |
| **T. Soups** | | | | |
| T1. All varieties of soups (includes broth) | 480 | 8 (1.7%) | 108 (22.5%) | 451 (94.0%) |
| **Category T Total** | **480** | **8 (1.7%)** | **108 (22.5%)** | **451 (94.0%)** |
| **U. Sugars & Sweets** | | | | |
| U1. Candies, confectionaries, chocolates | 541 | 480 (88.7%) | 372 (68.8%)^†^ | 0 |
| U3. Hard candies | 20 | 9 (45.0%) | 0 | 0 |
| U4. Baking candies | 47 | 41 (87.2%) | 47 (100.0%) | 0 |
| U5. Breath mints | 2 | 0 | 0 | 0 |
| U7. Icing sugar | 3 | 0 | 0 | 0 |
| U8. Honey, molasses, bread spreads | 72 | 14 (19.4%) | 5 (6.9%) | 0 |
| U9. Jams, jellies, fruit spreads | 228 | 205 (89.9%) | 2 (0.9%)^†^ | 0 |
| U10. Fruit leather | 20 | 20 (100.0%) | 1 (5.0%) | 0 |
| U11. Marshmallows | 12 | 12 (100.0%) | 0 | 0 |
| U12. Sugars | 45 | 0^†^ | 0^†^ | 0^†^ |
| U14. Syrups used as toppings | 52 | 0 | 0 | 0 |
| U15. Syrups used as ingredients | 20 | 0 | 0 | 0 |
| **Category U Total** | **1,062** | **781 (73.5%)** | **427 (40.2%)** | **0** |
| **V. Vegetables** | | | | |
| V1. Vegetables without sauce | 411 | 0 | 0^†^ | 10 (2.4%) |
| V2. Vegetables with sauce | 13 | 0 | 5 (38.5%) | 6 (46.2%) |
| V3. Vegetables used for garnishing/flavouring | 27 | 0^†^ | 0^†^ | 4 (14.8%) |
| V4. Chili pepper & green onion | 42 | 0 | 0^†^ | 24 (57.1%) |
| V5. Seaweed, dehydrated mushrooms | 19 | 0 | 0 | 6 (31.6%) |
| V6. Sprouts | 1 | 0 | 0 | 0 |
| V7. Vegetable juice and drink | 60 | 16 (26.7%) | 0 | 27 (45.0%) |
| V8. Olives | 65 | 0 | 2 (3.1%) | 61 (93.8%) |
| V9. Sun-dried tomatoes and other pickled or oil-packed vegetables | 172 | 5 (2.9%) | 1 (0.6%)^†^ | 104 (60.5%)0^†^ |
| V10. Relish | 15 | 1 (6.7%) | 0 | 4 (26.7%) |
| V11. Vegetable paste | 12 | 0 | 0 | 0 |
| V12. Vegetable sauce or purée | 34 | 0 | 0^†^ | 5 (14.7%) |
| **Category V Total** | **871** | **22 (2.5%)** | **8 (0.9%)** | **251 (28.8%)** |
| **W. Foods for <4 years old** | | | | |
| W1. Cereals to be prepared | 41 | 20 (48.8%) | 0^†^ | 0 |
| W2. Ready-to-eat cereals and cereal bars | 9 | 9 (100.0%) | 0^†^ | 0 |
| W3. Cookies, biscuits, etc. | 47 | 6 (12.8%) | 0^†^ | 0 |
| W4. Strained meat, desserts, combination dishes | 124 | 63 (50.8%) | 0^†^ | 0 |
| W5. Combination dishes | 9 | 0 | 0^†^ | 3 (33.3%) |
| W6. Juices | 2 | 2 (100.0%) | 0^†^ | 0 |
| **Category W Total** | **232** | **100 (43.1%)** | **0** | **3 (1.3%)** |
| **OVERALL TOTAL** | **17,521** | **4,709 (26.9%)** | **5,018 (28.6%)** | **5,527 (31.5%)** |

All values are presented as n (%); n=17,521. ^*^Major and minor subcategories were defined as per Health Canada’s Table of Reference Amounts for Foods[2]. ^†^Indicates categories with missing nutrient values. A total of 13 (0.1%), 299 (1.7%), and 11 (0.1%) products overall were missing values for total sugars, saturated fats, and sodium, respectively. Abbreviations: FLIP, Food Label Information Program; FOP, Front-of-Pack; TRA, Table of Reference Amounts for Foods.

# References

1. Government of Canada: Cannada Gazette, Part I, Vol 152, No. 6 Available from: <https://gazette.gc.ca/rp-pr/p1/2018/2018-02-10/pdf/g1-15206.pdf>. Accessed March 5 2020.

2. Health Canada.: Table of Reference Amounts for Food Available from: <https://www.canada.ca/en/health-canada/services/technical-documents-labelling-requirements/table-reference-amounts-food.html>. Accessed July 15 2019.
